# Mapping Genetic Regulation of Transcription to Identify Functional Variants and Genes Associated with Pancreatic Cancer Risk

**DOI:** 10.1002/advs.202517184

**Published:** 2026-03-13

**Authors:** Xiaoyang Wang, Hui Geng, Zhengyan Yao, Yuan Jiang, Can Chen, Zequn Lu, Shuangshuang Tian, Ming Zhang, Ruiyan Liu, Chenxi Feng, Bin Li, Xiaoping Miao, Jianbo Tian, Shaokai Zhang, Ying Zhu

**Affiliations:** ^1^ Department of Epidemiology and Biostatistics School of Public Health Wuhan University Wuhan China; ^2^ Department of Cancer Epidemiology The Affiliated Cancer Hospital of Zhengzhou University & Henan Cancer Hospital Zhengzhou China; ^3^ College of Public Health Zhengzhou University Zhengzhou China; ^4^ State Key Laboratory of Metabolism and Regulation in Complex Organisms TaiKang Center for Life and Medical Sciences, Wuhan University Wuhan China

**Keywords:** pancreatic cancer, expression quantitative trait loci, genome‐wide association studies, silencers, ST7L

## Abstract

Pancreatic cancer is one of the most lethal malignancies. Genome‐wide association studies (GWAS) identify multiple susceptibility loci, but most map to noncoding regions, leaving variant‐to‐gene links unresolved. In this study, a genome‐wide regulatory map is constructed using expression quantitative trait loci (eQTL) analysis of 482 pancreatic tissues, and integrated with a GWAS meta‐analysis to prioritize causal variants. A total of 82 significant variants and 15 target genes for pancreatic cancer risk are identified, with enrichment in cancer‐related pathways. The variant rs11102484 is validated in an independent cohort of 569 cases and 2691 controls. The combined analysis of 5699 cases and 8467 controls confirms that the G allele of rs11102484 significantly reduces pancreatic cancer risk (odds ratio = 0.85, 95% confidence interval = 0.80–0.90, *P* = 4.83 × 10^−8^). Functional assays demonstrate that the G allele impairs ZNF263 binding at rs11102484, thereby weakening a long‐range silencer‐promoter interaction and increasing *ST7L* expression. Elevated *ST7L* dampens AKT/β‐catenin signaling and suppresses pancreatic cancer cell proliferation, consistent with the protective association. Overall, this study implicates functional genes in pancreatic cancer risk and characterizes a regulatory variant that modulates *ST7L* expression, advancing the interpretation of GWAS findings and understanding of pancreatic cancer biology.

## Graphical abstract

Integration of a pancreatic eQTL map with a GWAS meta‐analysis identifies 82 putative functional variants and 15 genes. The association between rs11102484 and pancreatic cancer risk is observed in a total of 5699 cases and 8467 controls. The G allele of rs11102484 weakens ZNF263 binding and the silencer‐promoter interaction, thereby increasing *ST7L* expression and suppressing tumor cell proliferation.

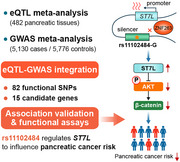

## 1 Introduction

Pancreatic cancer is one of the most lethal malignancies, with a five‐year survival rate below 10% [1]. Its poor prognosis is largely attributable to the absence of early symptoms and effective screening, often resulting in late‐stage diagnosis. In 2022, approximately 511 000 new cases and 467 000 deaths were reported globally, [2] highlighting its considerable disease burden. While several environmental and lifestyle risk factors have been implicated, [1] the underlying etiology, particularly the role of genetic susceptibility, remains incompletely understood. Elucidating the genetic basis of pancreatic cancer is crucial for identifying high‐risk individuals and improving early detection strategies.

Genome‐wide association studies (GWAS) have identified multiple risk loci associated with pancreatic cancer, [3, 4, 5, 6, 7, 8, 9] providing valuable insights into its genetic basis. However, the majority of these risk single nucleotide polymorphisms (SNPs) reside in non‐coding regions of the genome and are often in linkage disequilibrium (LD) with other SNPs, complicating the identification of causal variants and their functional interpretation. While a few studies have successfully linked specific GWAS loci [10, 11, 12, 13] such as chr5p15.33/*TERT* [10] and chr1p36.33/*KLHL17* [11] to biological mechanisms, the functional relevance of many other loci remains unclear. These challenges underscore the need for systematic approaches that integrate large‐scale datasets and functional assays to better characterize the biological significance of GWAS‐identified variants.

Expression quantitative trait loci (eQTL) analysis links non‐coding genetic variants to gene expression, helping to reveal the regulatory mechanisms underlying disease susceptibility. Several studies have integrated eQTL and GWAS data to identify potential pancreatic cancer susceptibility genes, [14, 15, 16, 17, 18] such as *PDX1*, [16, 17] *INHBA*, [16, 17] and *PDGFB*, [15] which are involved in critical biological processes and contribute to the understanding of the disease. However, previous efforts have often relied on pathway‐based approaches or relatively modest eQTL sample sizes, in part due to the limited availability of pancreatic tissue samples. Additionally, while statistical associations between candidate susceptibility genes and pancreatic cancer risk have been established, the functional relevance of many loci remains largely unexplored, as few studies have experimentally validated these associations. Therefore, larger‐scale studies that integrate comprehensive datasets, along with functional assays, are essential to validate the biological mechanisms behind identified risk variants and genes.

In this study, we aimed to connect pancreatic cancer risk variants to their regulatory targets and elucidate their functional consequences. To this end, we first conducted an eQTL meta‐analysis of 482 pancreatic tissue samples from TCGA and GTEx, which improved statistical power and expanded regulatory discovery across protein‐coding and non‐coding genes. We then integrated the eQTL data with GWAS results from Chinese and PanC4 populations to prioritize functional variants and candidate target genes associated with pancreatic cancer risk. Among these, rs11102484 showed the strongest functional evidence and was linked to suppression of tumorigenicity 7 like (*ST7L*) as its putative target gene. *ST7L* has been reported to act as a tumor suppressor in several cancer types and to inhibit cell proliferation through pathways such as AKT/β‐catenin signaling [19, 20, 21, 22]. However, its role and underlying mechanisms in pancreatic cancer remain poorly defined. We therefore investigated the rs11102484‐*ST7L* regulatory axis and demonstrated that the protective G allele attenuated a long‐range silencer‐promoter interaction, consequently upregulating *ST7L* expression. Elevated ST7L dampened AKT/β‐catenin signaling and suppressed pancreatic cancer cell proliferation, providing a mechanistic link between rs11102484 and reduced pancreatic cancer risk. Collectively, our study leverages eQTL meta‐analysis, GWAS, and functional assays to decipher the mechanisms of non‐coding risk variants and advance our understanding of pancreatic cancer etiology.

## 2 Results

### 2.1 Genome‐Wide Identification of eQTLs in Pancreatic Cancer and Normal Tissues

**FIGURE 1:**
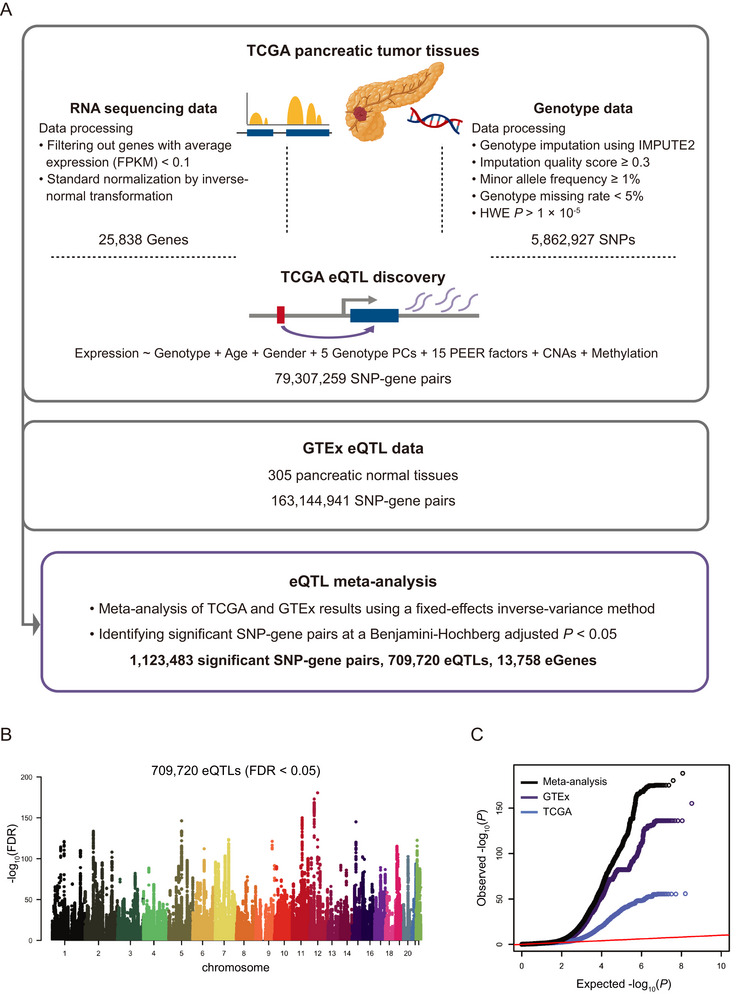
Overview of eQTL meta‐analysis in pancreatic cancer and normal tissues. A) Workflow of eQTL analysis. Cis‐eQTLs are mapped in TCGA pancreatic tumor tissues (*n* = 177) using a linear regression model with covariate adjustment, and summary statistics from GTEx normal pancreatic tissues (*n* = 305) are used. TCGA and GTEx results are combined using a fixed‐effects inverse‐variance meta‐analysis. Multiple testing is controlled using the BH procedure, and significant SNP‐gene pairs are defined at FDR < 0.05. B) Manhattan plot showing the genomic distribution of significant SNP‐gene pairs from the eQTL meta‐analysis (FDR < 0.05). C) Quantile‐Quantile plot of observed versus expected ‐log_10_(*P*) values for TCGA (blue), GTEx (purple), and the meta‐analysis (black).

To systematically investigate regulatory variants influencing gene expression, we conducted a genome‐wide cis‐eQTL meta‐analysis based on a total of 482 pancreatic tissue samples, including 177 tumor samples from TCGA and 305 normal samples from GTEx (Figure 1A). After data preprocessing and covariate adjustment, we identified 1 123 483 significant SNP‐gene pairs, corresponding to 709 720 unique eQTLs and 13 758 target genes (hereafter referred to as eGenes) at a false discovery rate (FDR) < 0.05 (Figure 1B). These eGenes included 10 304 protein‐coding genes, 2460 long non‐coding RNA (lncRNA) genes, 910 pseudogenes and 84 other gene types, reflecting the broad regulatory landscape captured by the meta‐analysis. Compared to single‐cohort analyses, the meta‐analysis markedly improved statistical power and enabled the identification of more eQTLs (Figure 1C). These findings provide a comprehensive resource for deciphering genetic regulatory mechanisms in both normal and malignant pancreatic tissues.

### 2.2 Regulatory Characteristics of eQTLs

**FIGURE 2:**
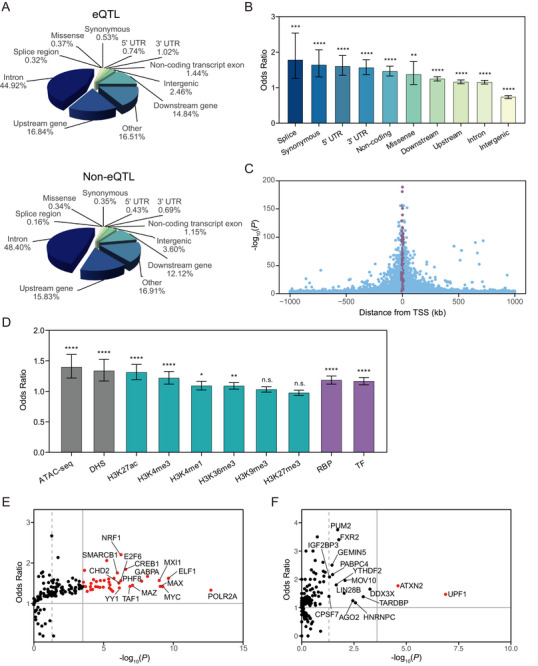
Functional characterization of eQTLs. A) Proportional distribution of eQTLs and non‐eQTLs across functional categories annotated by VEP. Percentages indicate the proportion of variants in each category. B) Enrichment of eQTLs across functional categories defined by VEP, compared to non‐eQTL variants. Error bars represent 95% CIs for ORs. C) Genomic distances between eQTLs (lead variants per gene) and corresponding eGene TSS. eQTLs within 10 kb of the TSS are shown in purple; distal eQTLs (≥10 kb) are shown in blue. D) Enrichment of eQTLs across regulatory features. Categories include open chromatin regions, histone modification marks, RBP binding sites, and TF binding sites. Error bars indicate 95% CIs for ORs. E) Enrichment of eQTLs in TF binding sites. Red dots indicate TFs with significant enrichment (*P*_Bonferroni_ < 0.05 and OR > 1); the top 15 TFs with the lowest *P* values are labeled. The horizontal gray line denotes OR = 1. The vertical dashed line indicates the nominal significance threshold (*p* = 0.05), and the solid line marks the Bonferroni‐corrected threshold. F) Enrichment of eQTLs in RBP binding sites. Labels denote RBPs with significant enrichment (OR > 1) at either nominal (*p* < 0.05) or Bonferroni‐corrected thresholds (*P*_Bonferroni_ < 0.05). Red dots indicate RBPs passing Bonferroni correction. The horizontal gray line represents OR = 1. The vertical dashed line marks the nominal significance threshold; the solid line marks the Bonferroni‐corrected threshold. Enrichment analyses in (B and D‐F) are evaluated using two‐sided Fisher's exact test. *P* values in (B and D) are adjusted using the BH method. n.s., not significant; ^*^ *p* < 0.05; ^**^ *p* < 0.01; ^***^ *p* < 0.001; ^****^ *p* < 0.0001.

We first annotated all significant eQTLs using the Variant Effect Predictor (VEP) to examine their distribution across genomic features. Compared to non‐eQTLs, eQTLs were significantly enriched in functional genomic regions, such as intronic regions and untranslated regions (5’ and 3’ UTRs), and were underrepresented in intergenic regions (Figure 2A,B). We then examined the genomic distribution of eQTLs relative to their target gene transcriptional start sites (TSSs). The strength of association peaked near the TSS and declined with distance (Figure 2C), indicating a spatial enrichment of regulatory variants in promoter‐proximal regions, consistent with previous observations [23].

Next, we assessed the enrichment of eQTLs across diverse regulatory features. Compared to matched non‐eQTLs, eQTLs showed significant enrichment in open chromatin regions (ATAC‐seq and DNase‐seq peaks) and activating histone marks, including H3K27ac, H3K4me1, and H3K4me3 (Figure 2D). A moderate enrichment was also observed for H3K36me3, whereas repressive marks such as H3K9me3 and H3K27me3 showed no significant enrichment. We further evaluated transcription factor (TF) binding sites and found that eQTLs were significantly enriched across a broad set of TFs (Figure 2D,E). Notably, enriched TFs included POLR2A, MYC, YY1, and ELF1, which have been reported to be implicated in pancreatic cancer and other malignancies [24, 25, 26, 27]. Similarly, eQTLs were significantly enriched in RNA‐binding protein (RBP) binding sites such as UPF1 and ATXN2 (Figure 2D,F), which have been previously linked to cancer‐related post‐transcriptional regulation [28, 29]. Together, these findings highlight that eQTLs are preferentially located in regions of gene regulation, exerting effects at both transcriptional and post‐transcriptional levels.

### 2.3 Biological and Clinical Relevance of eGenes in Pancreatic Cancer

**FIGURE 3:**
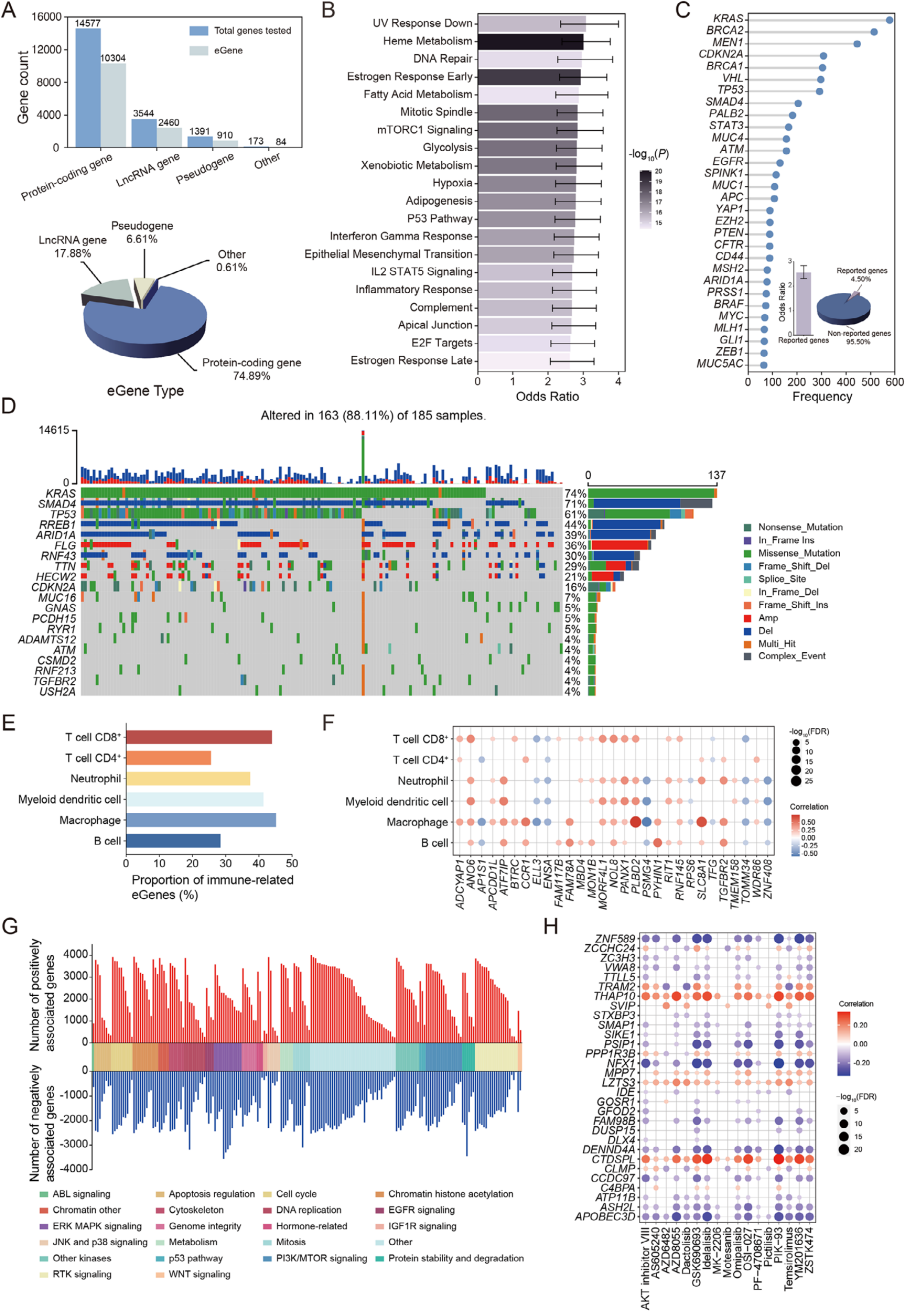
Biological and clinical characterization of eGenes. A) Gene biotype composition of all tested genes and eGenes. Numbers above bars indicate gene counts, and the pie chart shows the biotype distribution of eGenes. B) Pathway enrichment of eGenes in MSigDB Hallmark gene sets. The top 20 pathways are shown, ranked by ‐log_10_(*P*) value from two‐sided Fisher's exact test. Effect sizes are shown as ORs with 95% CIs (error bars). C) Enrichment of eGenes in frequently reported pancreatic cancer‐associated genes. The lollipop plot shows the top 30 genes ranked by reporting frequency. The bar plot below compares the enrichment of reported genes among eGenes relative to all background genes, based on two‐sided Fisher's exact test. Error bars indicate 95% CIs for ORs. The pie chart summarizes the proportion of reported versus unreported genes among eGenes. D) Somatic mutation and CNA landscape of the top 20 most frequently altered eGenes in TCGA pancreatic cancer samples (*n* = 185). Each column represents a tumor sample and each row represents an eGene. Mutation types and CNA events are indicated by color. E) Proportion of immune‐related eGenes in TCGA pancreatic cancer samples (*n* = 183). Immune infiltration levels are estimated using the TIMER algorithm. Immune‐related eGenes are defined as those showing significant associations between eGene expression and immune cell abundance with BH‐adjusted *p* value < 0.05. F) Correlations between eGene expression and immune cell infiltration in TCGA pancreatic cancer samples (*n* = 183). Each dot represents an eGene‐immune cell type pair. Spearman's rank correlation is used, and *p* values are adjusted using the BH method. Dot color indicates correlation direction (red for positive, blue for negative), and dot size reflects statistical significance (‐log_10_(FDR)). G) Significant eGene‐drug pairs identified based on the GDSC dataset. Associations between eGene expression and drug response are evaluated using Pearson correlation, and *p* values are adjusted using the BH method. Bars indicate positive (red) or negative (blue) associations between eGene expression and drug response. H) eGene‐drug associations for drugs targeting the PI3K/mTOR signaling pathway. Each dot represents a drug‐eGene pair. Dot color indicates correlation direction (red for positive, blue for negative); dot size reflects statistical significance (‐log_10_(FDR)).

A total of 13 758 eGenes were identified through eQTL meta‐analysis (Figure 3A). To investigate their functional relevance, we performed pathway enrichment analyses. Hallmark pathway analysis revealed that eGenes were significantly enriched in multiple cancer‐related pathways, including DNA repair, fatty acid metabolism, mTORC1 signaling, and the p53 pathway (Figure 3B). GO and KEGG enrichment analyses provided additional support for these findings (Figure S1). We further evaluated the enrichment of eGenes in a curated set of 1062 pancreatic cancer‐associated genes extracted from PubMed literature (Figure 3C). eGenes were significantly overrepresented in this set (*P* = 1.22 × 10^−66^), supporting their relevance to pancreatic cancer biology.

Next, we analyzed the somatic mutation and copy number alteration (CNA) profiles of eGenes in TCGA pancreatic cancer samples. The top 20 most frequently altered eGenes included well‐known tumor drivers and suppressors such as *KRAS* (74%), *SMAD4* (71%), *TP53* (61%), *RREB1* (44%), and *ARID1A* (39%) (Figure 3D). Somatic alterations such as missense mutations, deletions, and frameshift mutations were observed in these genes across 88.11% of pancreatic cancer samples, highlighting their broad involvement in tumorigenesis.

Given the critical role of the immune system in tumor progression and therapy response, we next examined the association between eGene expression and immune cell infiltration in pancreatic cancer. Using the TIMER algorithm, we found that a substantial proportion of eGenes were significantly correlated with the abundance of key immune cell populations, particularly macrophages, CD8^+^ T cells, and myeloid dendritic cells (Figure 3E). We further visualized the top immune‐related eGenes and their correlation patterns across six immune cell types (Figure 3F). Several eGenes exhibited strong associations with specific cell types. For example, *SLC8A1* expression was highly correlated with macrophage infiltration, whereas *ANO6* showed widespread correlations across multiple immune populations. These findings suggest that eGenes may contribute to the modulation of the tumor immune microenvironment through diverse regulatory mechanisms.

Finally, we explored the potential clinical relevance of eGenes in drug response by analyzing gene expression and drug sensitivity associations using the GDSC data. A large number of significant eGene‐drug pairs were identified (FDR < 0.05), spanning a wide range of therapeutic compounds and signaling pathways (Figure 3G). The PI3K/mTOR signaling accounted for the greatest number of drug‐associated eGenes. We then examined the top 30 eGenes associated with PI3K/mTOR‐targeting compounds, and observed strong positive or negative correlations with the sensitivity of multiple inhibitors such as PIK‐93, GSK690693, and ZSTK474 (Figure 3H). These findings suggest the potential of eGene expression profiles as biomarkers for predicting drug sensitivity and prioritizing targeted therapies in pancreatic cancer.

### 2.4 Prioritization of Functional SNPs and Susceptibility Genes Through eQTL‐GWAS Integration

**FIGURE 4:**
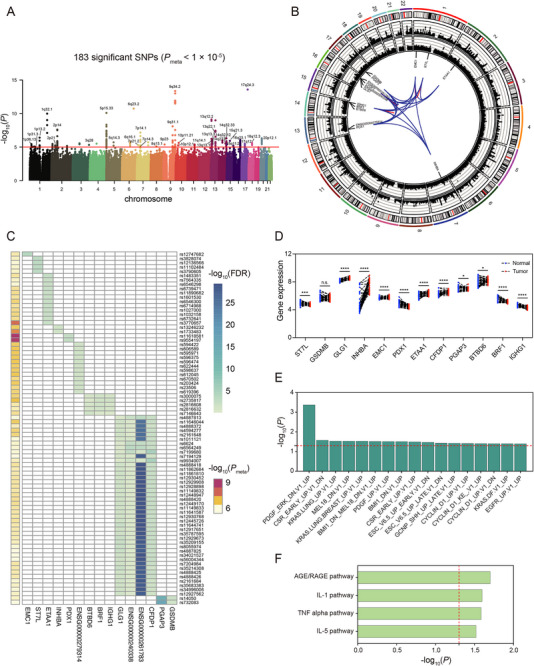
Integration of eQTL and GWAS data to identify candidate functional genes for pancreatic cancer. A) Manhattan plot of genetic variants associated with pancreatic cancer risk from GWAS meta‐analysis. The *x*‐axis indicates chromosomal positions and the *y*‐axis shows ‐log_10_(*P*). The red horizontal line marks the significance threshold (*P*_meta_ < 1 × 10^−5^). A total of 35 loci meeting *P*_meta_ < 1 × 10^−5^, *P*_CHN_ < 0.05, and *P*_PanC4_ < 0.05 are labeled. B) Circos diagram visualizing the integrative landscape of GWAS and eQTL results. From outermost to innermost: chromosomes, eQTL signals (FDR < 0.05), GWAS meta‐analysis signals (*P*_meta_ < 1 × 10^−5^), 15 candidate genes identified by the integrative analysis, and coexpression links among these genes (red: intra‐chromosomal; blue: inter‐chromosomal). C) Heatmap showing association statistics between 82 significant SNPs and 15 candidate genes. Each cell represents the ‐log_10_(FDR) of the eQTL association; the leftmost column shows ‐log_10_(*P*_meta_) values from GWAS for each SNP. D) Differential expression of candidate genes in pancreatic tumor versus adjacent normal tissues (GSE15471, *n* = 36 paired samples). The analysis is performed using limma with a paired design and empirical Bayes moderation, and *P* values are adjusted using the BH method to control FDR. n.s., not significant; ^*^FDR < 0.05; ^***^FDR < 0.001; ^****^FDR < 0.0001. E,F) Enrichment of candidate genes in MSigDB C6 oncogenic signature gene sets (E) and NetPath cancer signaling pathways (F). Significance is evaluated using the hypergeometric test. The red dashed line indicates the significance threshold (*p* = 0.05).

To systematically identify putative functional variants and susceptibility genes for pancreatic cancer, we integrated results from a GWAS meta‐analysis and the eQTL dataset. The GWAS meta‐analysis combined data from the PanC4 and Chinese cohorts, totaling 5130 cases and 5776 controls (Table S1). There was little evidence of genomic inflation in either the meta‐analysis (λ_meta_ = 1.045) or the individual studies (λ = 1.036–1.068) (Figure S2), supporting the reliability of the associations. The meta‐analysis identified 183 candidate SNPs across 35 genomic loci that were significantly associated with pancreatic cancer risk (*P*_meta_ < 1 × 10^−5^; *p* < 0.05 in both populations) (Figure 4A; Table S2).

By integrating these GWAS signals with the eQTL meta‐analysis, we prioritized 82 functional SNPs associated with both pancreatic cancer risk (*P*_meta_ < 1 × 10^−5^; *P*_CHN_ < 0.05; *P*_PanC4_ < 0.05) and gene expression (eQTL FDR < 0.05), mapping to 15 candidate genes (Figure 4B,C; Table S3). Heterogeneity metrics from the eQTL meta‐analysis for the 162 significant SNP‐gene pairs are provided in Table S4. Most pairs showed little heterogeneity between GTEx and TCGA, with only 11/162 (6.8%) exhibiting substantial heterogeneity (*I* ^2^ ≥ 75% and *P*_Het_ < 0.05). All 11 heterogeneous associations were significant in GTEx normal pancreas, whereas 9 of 11 were not significant in TCGA tumors. This discrepancy likely reflects bulk expression complexity in tumors and/or the smaller sample size of the TCGA cohort, which can attenuate detectable germline eQTL effects.

Notably, some candidate genes such as *ETAA1*, [30] *GSDMB*, [31] and *PDX1* [32] have been previously implicated in key cancer‐related processes, including DNA damage response, immune regulation, and pancreatic tumorigenesis, underscoring their relevance to pancreatic cancer biology. The 82 associated SNPs were located within nine genomic regions (1p13.2, 1p36.13, 2p14, 7p14.1, 13q12.2, 13q13.1, 14q32.33, 16q23.1, and 17q12), encompassing both known and potentially novel susceptibility loci. Together, these results shed light on the genetic basis of pancreatic cancer and offer biologically plausible candidates for functional studies.

### 2.5 Functional Annotation of Candidate Genes and Regulatory Variants

We evaluated the expression levels of candidate genes in the GSE15471 dataset, which includes 36 paired pancreatic tumor and adjacent normal tissue samples. Three genes (Ensembl IDs: ENSG00000279314, ENSG00000240338, and ENSG00000261783) were not available in the expression matrix, leaving 12 genes for differential expression analysis. *ST7L*, *PDX1*, *PGAP3*, *BRF1*, and *IGHG1* were significantly downregulated in tumor tissues, whereas *GLG1*, *INHBA*, *EMC1*, *ETAA1*, *CFDP1*, and *BTBD6* were significantly upregulated (Figure 4D). *GSDMB* showed no significant differential expression in this dataset. We then examined these genes in two additional GEO datasets (GSE28735 and GSE62165). Most genes showed directionally concordant tumor‐normal changes, while *ETAA1* and *IGHG1* were less consistent (Figure S3). *GSDMB* was consistently upregulated in both validation datasets, despite being non‐significant in GSE15471. To gain insights into the biological functions of this gene set, we conducted enrichment analyses using cancer‐related gene sets from the MSigDB C6 collection and signaling pathways from NetPath. The candidate genes were significantly enriched in oncogenic signature gene sets, which represent transcriptional responses associated with dysregulated cancer pathways (Figure 4E). In addition, they were also enriched in several cancer‐related signaling pathways, including AGE/RAGE, IL‐1, TNF‐α, and IL‐5 pathways (Figure 4F).

We further investigated the regulatory potential of the 82 candidate SNPs by integrating epigenetic annotation and computational prediction tools. All variants overlapped with at least one regulatory feature, including chromatin‐accessible regions or histone modification marks derived from pancreatic tissues or cancer cell lines (Table S5). Functional scores from RegulomeDB and CADD supported their potential regulatory impact. Among these, rs11102484 showed the strongest functional potential, marked by multiple histone modification peaks, a RegulomeDB score of 2b, and a CADD score of 9.64, and was therefore selected for downstream validation and mechanistic investigation.

### 2.6 Validation of the Association between rs11102484 and Pancreatic Cancer Risk

We next evaluated the association between rs11102484 and pancreatic cancer risk in an independent replication cohort consisting of 569 cases and 2691 controls (Table S6). In this replication analysis, rs11102484 remained significantly associated with pancreatic cancer risk, showing a consistent protective effect of the minor G allele [odds ratio (OR) = 0.81, 95% confidence interval (CI) = 0.71–0.92, *P* = 1.52 × 10^−3^], concordant with findings from the discovery GWAS meta‐analysis (OR = 0.86, 95% CI = 0.80–0.92, *P* = 6.48 × 10^−6^). A combined meta‐analysis incorporating all 5,699 cases and 8,467 controls further reinforced this association, with the G allele of rs11102484 conferring a significantly reduced risk of pancreatic cancer (OR = 0.85, 95% CI = 0.80–0.90, *P* = 4.83 × 10^−8^; Table 1). These results suggest that rs11102484 is a robust susceptibility variant for pancreatic cancer across populations.

**TABLE 1:** Association of rs11102484 with pancreatic cancer risk in GWAS meta‐analysis, replication and combined cohorts.

SNP	Cytoband	RA^a)^	EA^b)^	Study phase	OR^c)^ (95% CI^d)^)	*p* value
rs11102484	1p13.2	C	G	GWAS meta‐analysis	0.86 (0.80–0.92)	6.48 × 10^−6^
				Replication	0.81 (0.71–0.92)	1.52 × 10^−3^
				Combined	0.85 (0.80–0.90)	4.83 × 10^−8^

^a)^ RA: reference allele;

^b)^ EA: effect allele;

^c)^ OR: odds ratio;

^d)^ CI: confidence interval.

### 2.7 Allele‐Dependent Distal Silencer‐Promoter Interaction Linking rs11102484 to *ST7L* Regulation

**FIGURE 5:**
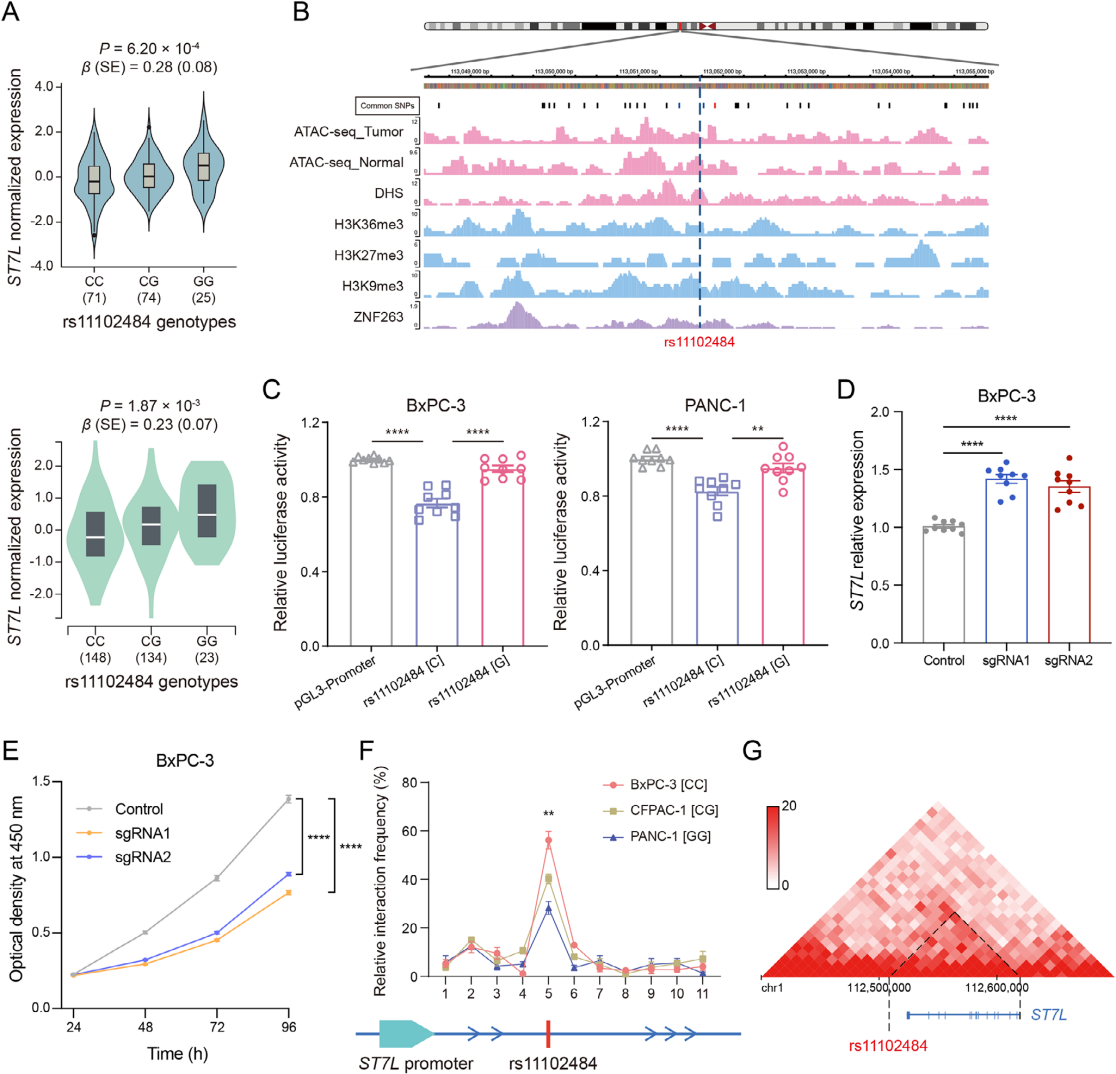
rs11102484 modulates *ST7L* expression through an allele‐dependent distal silencer‐promoter interaction. A) Association between rs11102484 genotypes and *ST7L* expression levels based on eQTL analyses in TCGA (with available genotype and expression data; *n* = 170) and GTEx (*n* = 305) pancreatic tissue datasets. B) Epigenetic marks at the rs11102484 locus. ATAC‐seq data from primary pancreatic tumor and adjacent normal tissues were generated and processed in our previous study. ChIP‐seq data for DHS, H3K36me3, H3K27me3, and H3K9me3 in pancreatic tissues were obtained from the Roadmap Epigenomics Project. ZNF263 ChIP‐seq data in K562 cells were retrieved from the Cistrome database (CistromeDB ID: 45958). The dashed line indicates the position of rs11102484. C) Relative luciferase activity of reporter constructs containing either the *C* or *G* allele of rs11102484 in BxPC‐3 and PANC‐1 cell lines. D) *ST7L* expression following CRISPRi‐mediated perturbation of the rs11102484‐containing element in BxPC‐3 cells. Relative *ST7L* mRNA levels are quantified by RT‐qPCR and normalized to the control group. Two independent sgRNAs (sgRNA1 and sgRNA2) are tested. E) Cell proliferation of BxPC‐3 cells following CRISPRi‐mediated perturbation of the rs11102484‐containing element, measured by CCK‐8 assay (OD_450_). F) 3C assays demonstrating allele‐specific chromatin interaction between the rs11102484‐containing region and the *ST7L* promoter across pancreatic cancer cell lines with distinct genotypes. G) Hi‐C interaction profile in the Capan‐1 pancreatic cancer cell line from the 3D Genome Browser, showing chromatin contact between the rs11102484 locus and the *ST7L* promoter region. Data in (C‐F) represent mean ± s.e.m. from *n* = 3 independent experiments, each performed with three technical replicates. Statistical significance is determined using a two‐tailed unpaired Student's *t*‐test (C‐E) or a one‐way ANOVA (F); ^**^ *p* < 0.01; ^****^ *p* < 0.0001.

To explore the regulatory role of rs11102484, we first examined its effect on *ST7L* expression. eQTL meta‐analysis showed that the minor G allele of rs11102484 was significantly associated with increased *ST7L* expression [*β* (SE) = 0.25 (0.05), FDR = 4.77 × 10^−4^], with consistent effects in both TCGA and GTEx pancreatic tissues (Figure 5A). Epigenomic annotation revealed that rs11102484 lay within a chromatin‐accessible region in both pancreatic tumor and adjacent normal tissues, as evidenced by ATAC‐seq data generated in our previous study (Figure 5B). The variant also overlapped with DNase I hypersensitive sites (DHSs) and multiple histone modification marks, including H3K36me3, H3K27me3, H3K9me3, H3K4me1, H3K4me3, and H3K27ac, according to the Roadmap Epigenomics Project (Figure 5B; Table S5). These features imply that the region harboring rs11102484 has regulatory potential.

We then performed dual‐luciferase reporter assays in BxPC‐3 and PANC‐1 cells to functionally validate the regulatory activity of the rs11102484‐containing region. The construct harboring the *C* allele exhibited significantly lower luciferase activity than the control vector, whereas the construct with the *G* allele showed markedly higher activity compared to the *C* allele construct (Figure 5C). These results indicate allele‐dependent regulatory activity and suggest a silencer‐like role. We next employed CRISPRi with guide RNAs targeting the rs11102484‐containing element in BxPC‐3 cells to directly confirm its silencer function. Both sgRNAs significantly increased *ST7L* expression compared with the control (Figure 5D). Consistently, CCK‐8 assays showed that CRISPRi‐mediated perturbation of this region suppressed cell proliferation in BxPC‐3 cells (Figure 5E).

The SNP rs11102484 is located approximately 112 kb downstream of the *ST7L* TSS, suggesting potential long‐range chromatin interactions between a distal regulatory element and the *ST7L* promoter. To investigate this possibility, we performed chromosome conformation capture (3C) assays in multiple pancreatic cancer cell lines with different rs11102484 genotypes. These assays revealed physical interaction between the rs11102484‐containing region and the *ST7L* promoter (Figure 5F). Interaction frequency was lowest in PANC‐1 cells (GG genotype), intermediate in CFPAC‐1 cells (CG genotype), and highest in BxPC‐3 cells (CC genotype), indicating an allele‐specific chromatin looping pattern. We further analyzed Hi‐C data from the Capan‐1 pancreatic cancer cell line using the 3D Genome Browser, and consistently observed spatial proximity between the rs11102484 region and the *ST7L* promoter (Figure 5G). These findings support a model in which rs11102484 influences *ST7L* expression by modulating long‐range silencer‐promoter interactions.

### 2.8 Reduced ZNF263 Binding to the rs11102484 G Allele Alleviates *ST7L* Transcriptional Repression

**FIGURE 6:**
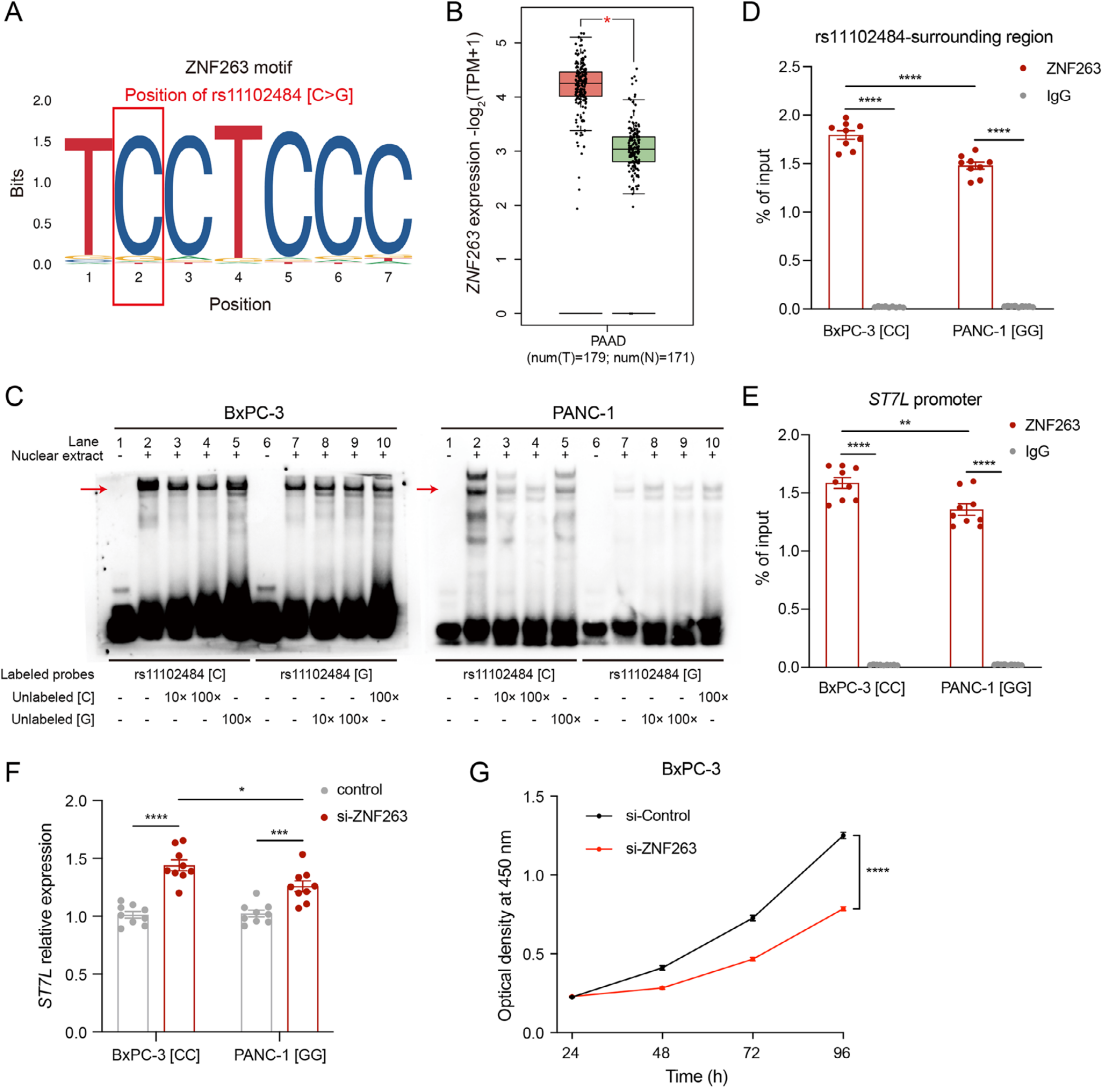
Allele‐specific ZNF263 binding to the rs11102484‐containing silencer regulates *ST7L* expression. A) ZNF263 binding motif disrupted by rs11102484 (C>G) based on the JASPAR database. B) Differential expression of *ZNF263* between pancreatic cancer (*n* = 179) and normal tissues (*n* = 171) from the GEPIA database. Red, tumor (T); green, normal (N). C) EMSAs using biotin‐labeled probes containing either the C or G allele of rs11102484, and nuclear extracts from BxPC‐3 and PANC‐1 cell lines. Red arrows indicate allele‐specific DNA‐protein complexes. 10 × and 100 × denote 10‐fold and 100‐fold excess of unlabeled competitors relative to the labeled probes, respectively. D) The allele‐specific binding of ZNF263 to the rs11102484‐containing region measured by ChIP‐qPCR in BxPC‐3 (CC genotype) and PANC‐1 (GG genotype) cell lines. E) ZNF263 binding to the *ST7L* promoter measured by ChIP‐qPCR in BxPC‐3 (CC genotype) and PANC‐1 (GG genotype) cell lines. F) *ST7L* expression following siRNA‐mediated *ZNF263* knockdown in BxPC‐3 (CC genotype) and PANC‐1 (GG genotype) cells. Relative *ST7L* mRNA levels are quantified by RT‐qPCR and normalized to the control group. G) Cell proliferation of BxPC‐3 cells following siRNA‐mediated *ZNF263* knockdown, measured by CCK‐8 assay (OD_450_). Data in (D‐G) represent mean ± s.e.m. from *n* = 3 independent experiments, each performed with three technical replicates. Statistical significance is determined using a two‐tailed unpaired Student's *t*‐test; ^*^ *p* < 0.05; ^**^ *p* < 0.01; ^***^ *p* < 0.001; ^****^ *p* < 0.0001.

We next sought to identify transcription factors that mediate the allele‐dependent silencer activity of the rs11102484‐containing element. RegulomeDB motif annotation highlighted two candidate factors overlapping the variant, ZNF263 and ZNF460 (Figure 6A; Figure S4). According to UniProtKB/Swiss‐Prot, ZNF263 is a well‐characterized transcriptional repressor, consistent with a repressive regulatory role at this locus, whereas ZNF460 is poorly characterized. GEPIA data further showed that *ZNF263* was significantly upregulated in pancreatic cancer (Figure 6B), whereas *ZNF460* exhibited lower overall expression with no significant differential expression (Figure S4). Accordingly, we prioritized ZNF263 for subsequent validation.

A representative public ZNF263 ChIP‐seq track showed occupancy signals at rs11102484 (Figure 5B). JASPAR motif scanning suggested preferential motif matching of ZNF263 to the *C*‐allele sequence, with reduced motif matching for the *G* allele (Figure 6A). We therefore performed electrophoretic mobility shift assays (EMSAs) using nuclear extracts from BxPC‐3 and PANC‐1 cells. In both cell lines, the probe containing the *C* allele formed stronger DNA‐protein complexes compared to the probe with the *G* allele (Figure 6C). Competitive binding assays further confirmed the allele specificity, as excess unlabeled *C* allele probes effectively competed with the labeled *C* probe in a dose‐dependent manner, whereas *G* allele probes did not show such competition. In addition, we validated this observation by ZNF263 ChIP‐qPCR in two pancreatic cancer cell lines with different rs11102484 genotypes. ZNF263 occupancy at the rs11102484‐containing region was lower in PANC‐1 (GG genotype) cells than in BxPC‐3 (CC genotype) cells (Figure 6D), indicating reduced ZNF263 binding to the *G* allele.

Given the long‐range silencer‐promoter interaction between the rs11102484‐containing element and *ST7L*, we next asked whether ZNF263 was also recruited to the *ST7L* promoter in an allele‐dependent manner. To this end, we performed ZNF263 ChIP‐qPCR at the *ST7L* promoter in pancreatic cancer cell lines. The promoter region showed significant enrichment in ZNF263 immunoprecipitates compared with the IgG control, with lower promoter binding in PANC‐1 (GG genotype) cells than in BxPC‐3 (CC genotype) cells (Figure 6E). These results demonstrate genotype‐associated ZNF263 occupancy at the *ST7L* promoter. Functionally, siRNA‐mediated knockdown of *ZNF263* increased *ST7L* mRNA expression in both BxPC‐3 (CC genotype) and PANC‐1 (GG genotype) cells (Figure 6F; Figure S5), supporting a negative regulatory role of ZNF263 on *ST7L* transcription. The induction was smaller in PANC‐1 than in BxPC‐3, consistent with diminished ZNF263 binding to the rs11102484 G allele. CCK‐8 assays further revealed that *ZNF263* knockdown substantially suppressed cell proliferation in BxPC‐3 cells (Figure 6G). Together, these results reveal that the rs11102484 G allele reduces ZNF263 binding and thereby alleviates transcriptional repression of *ST7L*.

### 2.9 Tumor Suppressor Role of *ST7L* in Pancreatic Cancer

**FIGURE 7:**
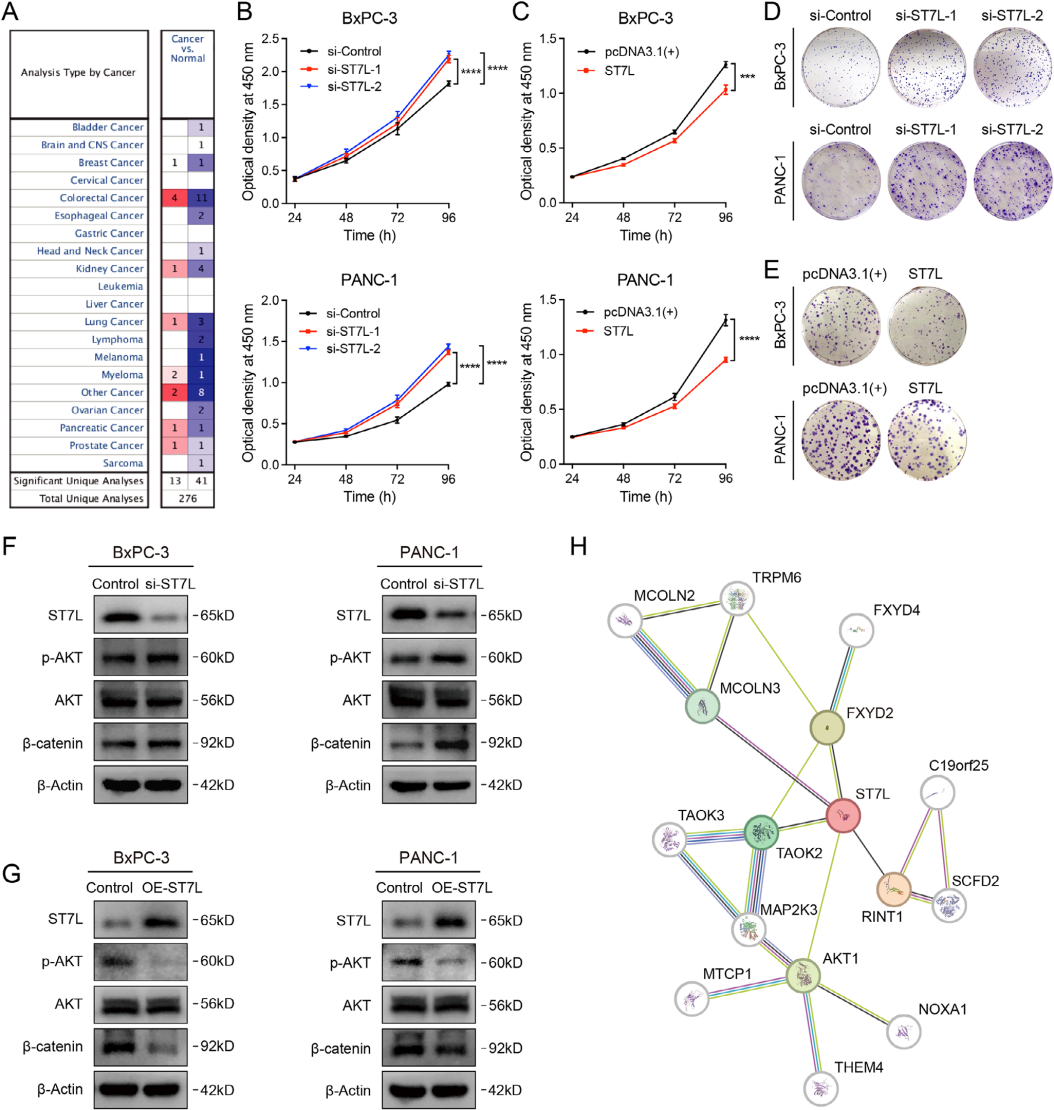
ST7L exerts tumor‐suppressive effects in pancreatic cancer by suppressing AKT/β‐catenin signaling. A) Summary of *ST7L* expression across multiple cancer types based on the Oncomine database. The heatmap shows the number of significant differential expression analyses (cancer vs. normal) by cancer type, as defined in Oncomine. Red indicates higher expression in cancer tissues; blue indicates lower expression in cancer tissues. B,C) Cell proliferation of BxPC‐3 and PANC‐1 cell lines after *ST7L* knockdown (B) or overexpression (C), as measured by CCK‐8 assays (OD_450_). Data represent mean ± s.e.m. from *n* = 3 independent experiments, each performed with three technical replicates. Statistical significance was determined using a two‐tailed unpaired Student's *t*‐test; ^***^ *p* < 0.001; ^****^ *p* < 0.0001. D,E) Colony formation assays in BxPC‐3 and PANC‐1 cell lines after *ST7L* knockdown (D) or overexpression (E). Images are representative of *n* = 3 independent experiments. F,G) Western blot analysis of ST7L, p‐AKT (Ser473), total AKT, and β‐catenin in BxPC‐3 and PANC‐1 cells following *ST7L* knockdown (F) or overexpression (G). β‐actin is used as a loading control. H) PPI network of ST7L predicted by the STRING database. The network is constructed with medium confidence (score ≥ 0.4), displaying ST7L and its first‐ and second‐shell interactors.

To investigate the potential role of *ST7L* in pancreatic cancer, we first assessed its expression in tumor versus normal tissues. *ST7L* was consistently downregulated in pancreatic cancer samples across three independent GEO cohorts (Figure 4D; Figure S3). Oncomine‐derived expression results further showed broadly decreased *ST7L* expression across multiple cancer types (Figure 7A). To further define the cellular context of *ST7L* expression, we analyzed a single‐nucleus RNA‐seq (snRNA‐seq) dataset (GSE202051) and a single‐cell RNA‐seq (scRNA‐seq) dataset (CRA001160). *ST7L* showed overall low expression at single‐cell resolution in pancreatic cancer. Detectable transcripts were primarily observed in epithelial cells, fibroblasts and endothelial cells (Figure S6A,B). Curated pancreatic cancer datasets from TISCH2 showed a similar pattern, with *ST7L* expression relatively enriched in epithelial and fibroblast clusters compared with immune cell populations (Figure S6C).

We then explored the clinical relevance of *ST7L* by examining its associations with patient survival and drug sensitivity. In the TCGA pancreatic cancer cohort, patients with higher *ST7L* expression tended to have longer overall survival (hazard ratio = 0.68, log‐rank *p* = 0.065) and showed significantly improved progression‐free survival (hazard ratio = 0.66, log‐rank *p* = 0.023) compared with the low‐expression group (Figure S7A,B). Drug sensitivity results retrieved from the GSCA platform indicated that *ST7L* expression was significantly correlated with the sensitivity of several agents in cancer cell lines, such as A‐770041, dexamethasone, and sorafenib (Figure S7C,D).

To experimentally evaluate the functional relevance of *ST7L* in tumor cell growth, we performed CCK‐8 and colony formation assays in BxPC‐3 and PANC‐1 cells following *ST7L* knockdown or overexpression (Figure S8). *ST7L* knockdown substantially promoted cell proliferation (Figure 7B), whereas its overexpression significantly suppressed proliferation (Figure 7C). In agreement with these results, colony formation assays showed that knockdown of *ST7L* enhanced colony formation ability, while its overexpression markedly reduced the number of colonies formed in both cell lines (Figure 7D,E).

### 2.10 ST7L Attenuates AKT/β‐Catenin Signaling in Pancreatic Cancer Cells

ST7L has been implicated in β‐catenin signaling in several cancers [19, 20, 21] and reported to suppress the AKT/GSK3β/β‐catenin axis in hepatocellular carcinoma [21]. To determine whether ST7L modulates the AKT/β‐catenin pathway in pancreatic cancer, we performed western blot analysis following *ST7L* knockdown or overexpression. Compared with the control, siRNA‐mediated *ST7L* knockdown led to a marked increase in AKT phosphorylation at Ser473, accompanied by a substantial elevation of β‐catenin levels in both BxPC‐3 and PANC‐1 cells (Figure 7F). Conversely, *ST7L* overexpression reduced p‐AKT (Ser473) and β‐catenin levels in both cell lines (Figure 7G). These findings suggest that ST7L may exert tumor‐suppressive effects in pancreatic cancer by inhibiting AKT/β‐catenin signaling.

To further explore the molecular context of ST7L, we constructed a protein‐protein interaction (PPI) network using the STRING database (Figure 7H). The network identified multiple predicted first‐ and second‐shell interactors of ST7L, implicating its potential involvement in diverse signaling pathways. In addition to AKT1, linked nodes included TAOK2 and MAP2K3, which are involved in MAPK signaling cascades. RINT1 was also highlighted as a predicted interactor, with reported roles in cell cycle checkpoint regulation and telomere length control [33, 34]. Overall, this PPI analysis nominates functional modules associated with ST7L, complementing our targeted validation and suggesting avenues for future mechanistic dissection.

## 3 Discussion

**FIGURE 8:**
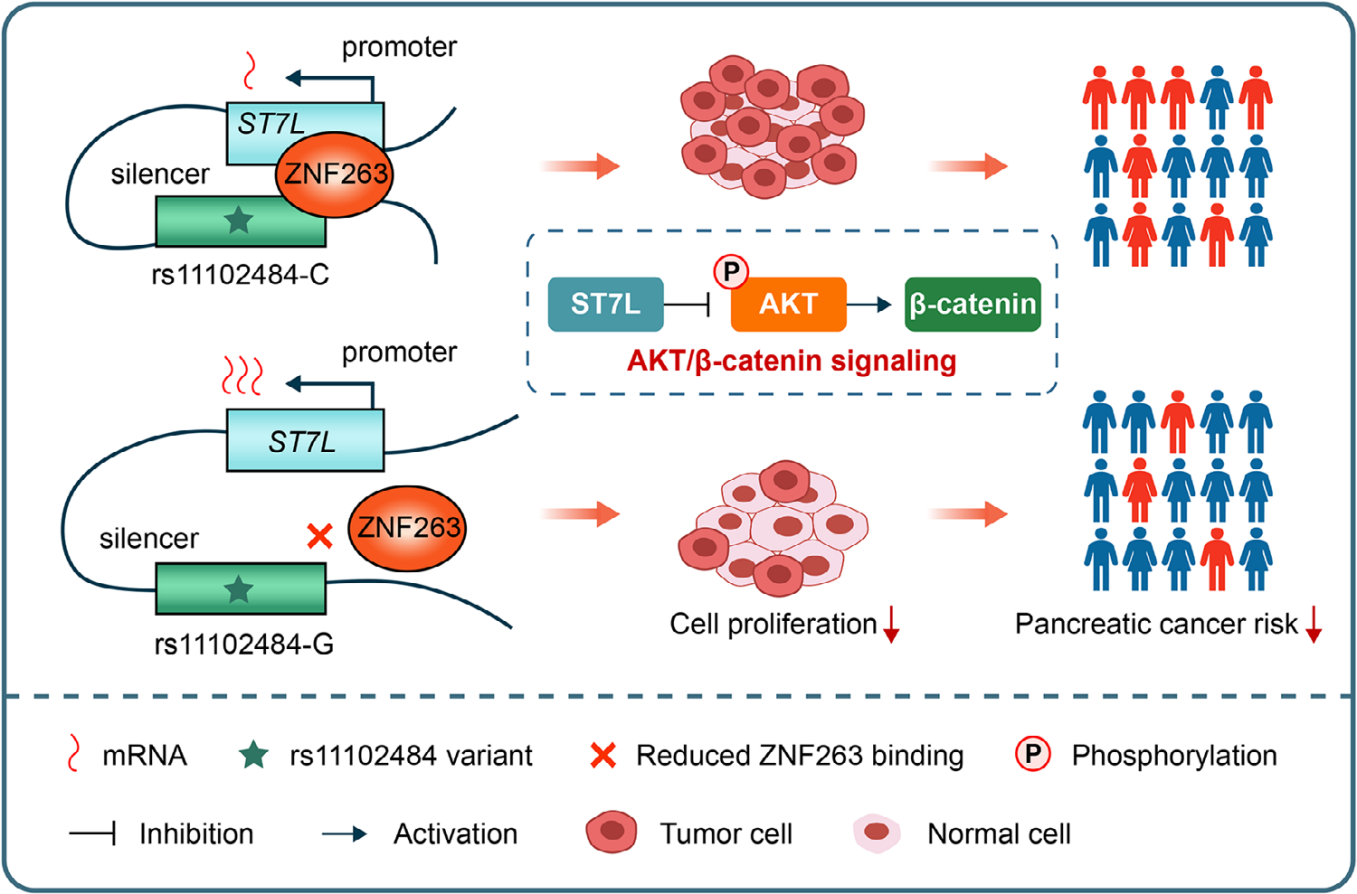
Schematic illustrating the role of rs11102484 in pancreatic cancer risk. The *C* to *G* change at rs11102484 reduces ZNF263 binding at a silencer element and attenuates long‐range silencer‐promoter interaction, resulting in higher *ST7L* expression. Increased ST7L suppresses AKT phosphorylation and β‐catenin signaling, leading to reduced cell proliferation and lower pancreatic cancer susceptibility. Symbols are explained in the inset legend.

In this study, we performed a genome‐wide investigation of regulatory associations using 482 pancreatic tissue samples from TCGA and GTEx, and integrated these findings with GWAS results. This analysis identified 82 putative functional SNPs and 15 candidate genes associated with pancreatic cancer risk. These variants overlapped regulatory features such as chromatin‐accessible regions and histone modification marks, and the candidate genes were enriched in cancer‐related signaling pathways. Through replication analyses and functional assays, we pinpointed rs11102484 as a regulatory susceptibility variant that modulated *ST7L* expression via a ZNF263‐associated silencer mechanism (Figure 8). This integrative‐to‐functional strategy bridges genetic association and mechanistic insights, strengthening the interpretability of the findings.

To maximize power to detect genetic influences on baseline pancreatic gene expression, we combined GTEx and TCGA eQTL data in a fixed‐effects meta‐analysis, which increased the sample size and helped to prioritize stable germline signals for downstream integration with GWAS. This strategy also provided a more comprehensive view of the pancreatic regulatory landscape, extending the analysis to non‐coding genes such as lncRNAs. While our meta‐analysis focuses on shared germline effects, identification of tumor‐specific eQTLs is an important direction for future work and will require larger, harmonized tumor and normal cohorts to characterize regulatory changes arising during pancreatic tumorigenesis. In addition, we observed that the eGenes were linked to recurrent somatic alterations, immune infiltration associations, and broad drug‐sensitivity correlations, underscoring their potential biological and translational relevance in pancreatic cancer.

We further evaluated heterogeneity for the 162 SNP‐gene pairs from GWAS‐eQTL integration. The vast majority of pairs exhibited concordant effects across GTEx and TCGA, supporting a predominantly germline regulatory basis for these associations. Only a small proportion (11/162, 6.8%) showed substantial heterogeneity, which was mainly characterized by attenuation in TCGA tumors compared with GTEx normal pancreas. This heterogeneity may reflect bulk tumor confounding (e.g., cellular composition and tumor purity) and/or the smaller sample size of the TCGA tumor cohort. Accordingly, this heterogeneous subset warrants validation in larger independent datasets and cell type‐resolved studies.

A total of 15 putative functional genes for pancreatic cancer were identified in our integrative study, encompassing both previously reported and newly prioritized candidates. These genes were significantly enriched in oncogenic signature gene sets and key signaling pathways, including AGE/RAGE, IL‐1, TNF‐α, and IL‐5 pathways. Most prioritized genes showed directionally concordant tumor‐normal expression changes across three independent GEO cohorts, with only limited dataset‐dependent variability. This variability may arise from differences in cohort characteristics, control definitions, and analytic pipelines. Notably, seven of the 15 candidate genes (*GSDMB*, *PDX1*, *PGAP3*, *CFDP1*, *BTBD6*, *INHBA*, and *ETAA1*) have been reported in previous GWAS or transcriptome‐wide association studies in pancreatic cancer [5, 6, 16, 17]. Among them, *PDX1* encodes a transcriptional activator essential for pancreatic development. It may exert tumor‐suppressive effects early in tumorigenesis but acquire pro‐tumorigenic functions during progression, [35] with elevated expression shown to enhance malignant phenotypes of pancreatic cancer cells [36, 37, 38]. *GSDMB* has been implicated in antitumor immunity by enabling granzyme A (GZMA)‐mediated pyroptosis of tumor cells, thereby potentially shaping immune surveillance and responses to immune checkpoint blockade [31]. *ETAA1*, which has been linked to pancreatic cancer susceptibility, functions in replication‐stress responses by cooperating with RPA to stabilize stalled replication forks [30]. These findings highlight the biological significance of the identified targets and validate the effectiveness of this integrative approach in prioritizing functional susceptibility genes.

We pinpointed *ST7L* as a key novel target gene of rs11102484, a susceptibility variant located in a silencer region at 1p13.2. *ST7L* has been reported to be downregulated in various cancers, including cervical cancer and breast cancer [20, 22]. Our differential expression analyses revealed significantly lower expression of *ST7L* in pancreatic tumor tissues compared to normal tissues, corroborating previous immunohistochemical observations in pancreatic cancer [39]. Functional assays demonstrated that *ST7L* inhibited cell proliferation in both BxPC‐3 and PANC‐1 cells, consistent with prior reports of *ST7L*‐mediated suppression of proliferation, migration, and invasion across multiple cancer types [19, 20, 21, 22]. Mechanistically, ST7L has been implicated in β‐catenin signaling in several malignancies and reported to interact with AKT in hepatocellular carcinoma [19, 20, 21]. However, its role in pancreatic cancer has remained unclear. This study showed that *ST7L* knockdown increased p‐AKT and β‐catenin levels in pancreatic cancer cells, while *ST7L* overexpression exerted an opposite effect. The PPI analysis nominated additional candidate nodes associated with ST7L (e.g., RINT1, TAOK2, and MAP2K3), which are involved in key pathways regulating cancer cell growth, survival, and metabolism. These results suggest that ST7L may exert tumor‐suppressive effects by dampening AKT/β‐catenin signaling and linking to broader cancer‐related signaling pathways. Notably, the PPI network highlights putative ST7L‐associated partners, and whether ST7L modulates AKT/β‐catenin signaling through direct protein interaction or indirect intermediate regulators warrants further investigation. Interaction assays such as co‐immunoprecipitation will be valuable to test physical interactions and delineate the ST7L‐centered signaling cascade. Given the pronounced heterogeneity of pancreatic cancer, validation in multi‐stage patient cohorts and organoid or co‐culture models is also needed to further refine how tumor stage and microenvironment shape ST7L‐dependent signaling. Taken together, these mechanistic insights, along with our exploratory survival and drug sensitivity analyses, strengthen the clinical relevance of *ST7L* in pancreatic cancer and support its translational promise as a tumor‐suppressive regulator.

Importantly, we complemented the integrative prioritization with independent association replication and functional validation for rs11102484, establishing its regulatory role in pancreatic cancer. The rs11102484 G allele increased *ST7L* expression by attenuating ZNF263‐associated silencer activity and weakening the long‐range silencer‐promoter interaction, offering a mechanistic explanation for its protective association with pancreatic cancer susceptibility. While our data validated ZNF263 as a central repressive factor at this locus, we cannot fully exclude contributions from additional TFs such as ZNF460 or co‐regulators acting in the same regulatory node. Future studies using more comprehensive TF perturbation experiments will help refine the regulatory hierarchy.

Several limitations of this study should be considered. First, we used a suggestive GWAS threshold (*P*_meta_ < 1 × 10^−5^) in the discovery phase to maximize the capture of promising SNPs for integrative analyses. This less stringent criterion may increase the false‐positive rate. Therefore, we prioritized loci supported by eQTL evidence and carried forward rs11102484 for validation in an independent cohort. The association was replicated and reached genome‐wide significance (*P* < 5 × 10^−8^) in the combined analysis. Nevertheless, comprehensive replication and experimental validation of other candidate SNPs will be required to clarify the biological mechanisms. Second, the eQTL resources used in this study were derived from TCGA and GTEx, which consist largely of individuals of European ancestry and therefore provide limited ethnic diversity. The subsequent integrative analyses combined these eQTL data with cross‐ancestry GWAS data from Han Chinese and PanC4 cohorts, which may influence local LD structure and consequently affect colocalization and causal variant mapping. To improve the generalizability of the findings, future studies incorporating both eQTL and GWAS datasets from more diverse populations would be beneficial. Third, our eQTL analyses were performed in bulk pancreatic tissues, where variation in epithelial, stromal, and immune cell composition across samples may confound regulatory associations. Thus, the observed eQTL signals represent average effects across mixed cell populations rather than cell type‐resolved mechanisms. Developing single‐cell eQTL maps in pancreatic cancer will be required to delineate cell type‐specific regulatory effects.

Overall, we systematically integrated eQTL and GWAS data to map germline regulatory variants underlying pancreatic cancer susceptibility. While most signals appear germline‐driven and robust, the small heterogeneous subset may point to tumor‐specific regulatory mechanisms that should be pursued in future work at single‐cell resolution. Our integrative analysis prioritizes a set of putative functional variants and target genes, highlighting the rs11102484‐*ST7L* regulatory axis in pancreatic cancer risk. These findings offer new insights into the genetic underpinnings of pancreatic cancer and provide a focused resource for future mechanistic and translational research.

## 4 Methods

### 4.1 Data Acquisition

To identify regulatory variants in pancreatic cancer, we obtained RNA‐seq (level 3, Illumina HiSeq 2000), somatic CNAs (Affymetrix Genome‐Wide Human SNP Array 6.0), DNA methylation (Illumina Infinium Human DNA Methylation 450), and SNP genotyping data (Affymetrix Genome‐Wide Human SNP Array 6.0) from TCGA. Additionally, cis‐eQTL summary statistics for 305 normal pancreatic tissues were downloaded from GTEx v8.

### 4.2 Data Processing

For TCGA samples (*n* = 177), gene expression levels were quantified as the fragments per kilobase of transcript per million mapped reads (FPKM). To reduce the effect of outliers, we filtered out genes with average FPKM < 0.1 and applied an inverse‐normal transformation to gene expression matrix [40, 41]. Genotype data were imputed using IMPUTE2 with the 1000 Genomes Project (Phase 3) as the reference panel. Post‐imputation quality control retained autosomal variants with minor allele frequency (MAF) ≥ 1%, genotype missing rate < 5%, Hardy Weinberg Equilibrium (HWE) *P* > 1 × 10^−5^ and imputation quality score (INFO score) ≥ 0.3.

### 4.3 eQTL Analysis

Prior to eQTL mapping in TCGA pancreatic cancer samples, we performed principal component analysis to estimate the effects of population genetic structure. We then used probabilistic estimation of expression residuals (PEER) [42] to compute confounding influences including batch effects on gene expression. After that, cis‐eQTL mapping (±1 Mb from gene TSS) was conducted with the MatrixEQTL R package using a linear regression model, adjusting for covariates including age, gender, the top 5 ancestry principal components (PCs), the top 15 PEER factors, CNAs and DNA methylation.

To maximize power to detect germline regulatory effects, we combined eQTL results from TCGA and GTEx and performed a fixed‐effects inverse‐variance meta‐analysis using the meta R package, based on effect sizes (*β*) and *P* values. The combined *P* values were corrected using the Benjamini–Hochberg (BH) procedure, and significant SNP‐gene pairs were identified at FDR < 0.05. Study heterogeneity was assessed using Cochran's Q test and quantified by *I* ^2^.

### 4.4 Functional Annotation and Enrichment Analysis of eQTLs

Genomic annotations were obtained using VEP. Epigenomic features, including histone modifications, DHSs, and chromatin accessibility data from human pancreatic normal tissues or cancer cell lines, were obtained from the Roadmap Epigenomics Project and Cistrome Data Browser. TF binding site data were downloaded from ENCODE TFBS clusters (v3), and RBP data were obtained from POSTAR3 (CLIPdb).

For enrichment analysis, the most significant eQTLs per gene were selected as the representative eQTL set. A matched set of non‐eQTLs (FDR ≥ 0.05) of equal size was sampled, controlling for MAF and distance to the TSS of the corresponding gene [43]. Enrichment was evaluated by comparing eQTLs and non‐eQTLs using Fisher's exact test. ORs and 95% CIs were calculated. *P* values were adjusted using the BH procedure for enrichments across VEP functional categories and histone mark annotations, and Bonferroni correction was applied when testing enrichment across multiple TFs and RBPs. An FDR or Bonferroni‐adjusted *p* value < 0.05 was considered statistically significant.

### 4.5 Functional and Clinical Annotation of eGenes

To characterize the biological and clinical relevance of eGenes, we performed gene set enrichment, literature mining, genomic alteration, immune association, and drug response analyses. Hallmark pathway enrichment was conducted using the “H: hallmark gene sets” from MSigDB, with Fisher's exact test to compare eGenes against all background genes. GO and KEGG enrichment analyses were performed using the R package clusterProfiler. Pancreatic cancer‐associated genes were extracted from PubMed abstracts published between 1990/01/01 and 2023/12/31 using disease‐ and mechanism‐related keywords. A total of 1062 genes with a term frequency of at least five were retained and used to assess enrichment among eGenes. Immune cell infiltration estimates were derived from TCGA expression data using TIMER, and associations between eGene expression and immune cell abundance were assessed using two‐sided Spearman's rank correlation with BH correction. Drug sensitivity (IC50) data were obtained from the GDSC resource, and gene‐drug associations were evaluated using two‐sided Pearson correlation with BH correction. Associations with FDR < 0.05 were considered statistically significant.

### 4.6 Study Populations

Two GWASs contributed a total of 10 906 samples to the meta‐analysis after quality control. Specifically, 981 pancreatic cancer cases and 1991 controls of Han Chinese ancestry were included from our previous GWAS study [4]. An additional 4149 cases and 3785 controls were obtained from nine studies within the PanC4. Recruitment details for all contributing studies have been described previously [4, 6]. All participants provided written informed consent, and all studies received ethical approval from their respective institutional review boards.

The GWAS sample size was determined by the number of cases and controls available after quality control. Statistical power was estimated using the GAS Power Calculator under an additive model. A two‐sided significance level of α = 1 × 10^−5^ was used, which was the suggestive threshold applied in this study. Representative allele frequency and effect size parameters were assumed (MAF = 0.20 and OR = 1.20). Under these assumptions, the discovery cohort including 5130 cases and 5776 controls achieved approximately 93.4% power to detect the specified genetic effect.

To replicate the association signals of candidate SNPs, we recruited an independent cohort of 569 pancreatic cancer cases and 2691 controls from Tongji Hospital and Union Hospital, affiliated with Tongji Medical College of Huazhong University of Science and Technology (Wuhan, China). All cases were histopathologically confirmed, and controls were cancer‐free individuals selected during routine physical examinations. Ethical approval was obtained from the Clinical Research Institution Review Committee and Ethics Review Committee of Wuhan University (number: WHU‐LFMD‐IRB2023003).

### 4.7 Genotyping and Imputation

The Chinese GWAS samples were genotyped using the Affymetrix GeneChip Human Mapping 6.0 set, while the PanC4 GWAS samples were genotyped using the Illumina HumanOmniExpressExome‐8v1 array. Replication‐stage genotyping was performed using TaqMan SNP genotyping assays on an ABI 7900HT system (Applied Biosystems)

Standard GWAS quality control procedures were applied. Briefly, unexpected duplicates and first‐ or second‐degree relatives were excluded. SNPs were filtered based on the following criteria: non‐autosomal location, call rate < 95%, MAF < 1%, or deviation from HWE (*P* < 1 × 10^−5^). Genotype imputation was conducted using the Michigan Imputation Server. The Chinese GWAS samples were imputed using the 1000 Genomes Project Phase 3 reference panel, and the PanC4 samples were imputed with the Haplotype Reference Consortium (HRC) panel. Post‐imputation, SNPs with an imputation quality score (Rsq) < 0.3 were excluded from downstream analyses.

### 4.8 GWAS Meta‐Analysis

Primary association analyses of SNPs with pancreatic cancer risk were conducted separately in each GWAS using logistic regression under an additive genetic model in PLINK v1.90, adjusting for age, gender, and the top three PCs of ancestry. The results from each study were then combined using a fixed‐effects inverse‐variance meta‐analysis in META v1.7. Cochran's Q test and *I* ^2^ statistic were used to assess heterogeneity between studies. To maximize discovery of promising candidates, loci showing suggestive significance (*P*_meta_ < 1 × 10^−5^) and nominal association in both PanC4 and Chinese datasets (*p* < 0.05 in each) were selected for downstream integrative and functional analyses. As this more permissive threshold may increase false positives, we performed independent replication and functional assays to validate the prioritized variant.

### 4.9 Integration of eQTL and GWAS Data and Functional Analyses

To identify putative regulatory SNPs involved in pancreatic cancer, we integrated GWAS and eQTL data by selecting SNP‐gene pairs that showed evidence of association in both datasets. Specifically, pairs with genome‐wide suggestive significance in the GWAS meta‐analysis (*P*_meta_ < 1 × 10^−5^, *P*_PanC4_ < 0.05 and *P*_CHN_ < 0.05) and eQTL significance (FDR < 0.05) were retained.

To functionally characterize the prioritized candidate genes, we performed differential expression analyses in three GEO datasets. GSE15471 [44] and GSE28735 [45, 46] include paired pancreatic tumor and adjacent control tissues (*n* = 36 pairs and *n* = 45 pairs, respectively). GSE62165 [47] comprises pancreatic tumor (*n* = 118) and normal (*n* = 13) tissues. Differential expression was analyzed using the limma package with empirical Bayes moderation. Paired designs were used for GSE15471 and GSE28735 to account for within‐patient pairing, whereas a two‐group model was used for GSE62165. *P* values were adjusted using the BH method. Pathway enrichment analyses were conducted using oncogenic signature gene sets from the MSigDB C6 collection and curated cancer signaling pathways from NetPath. Enrichment was assessed using the hypergeometric test.

For the integrative functional annotation of candidate SNPs, we examined their overlap with regulatory elements, including chromatin‐accessible regions (ATAC‐seq and DNase‐seq peaks) and histone modification marks (H3K4me3, H3K4me1, H3K27ac, H3K27me3, H3K9me3, and H3K36me3). In addition, multiple bioinformatics tools, including RegulomeDB (hg19), CADD (GRCh37‐v1.6), and HaploReg, were employed to further characterize the regulatory potential of these variants.

### 4.10 Regulatory Annotation of the rs11102484 Region

Genome tracks around rs11102484 were visualized in the Integrative Genomics Viewer (IGV). Chromatin accessibility was assessed using our ATAC‐seq data from a matched pair of pancreatic tumor and adjacent normal tissues. DNase‐seq peaks and histone mark ChIP‐seq peaks in pancreatic tissues were retrieved from the Roadmap Epigenomics Project. TF motifs overlapping rs11102484 were queried using RegulomeDB, and allele‐dependent motif scanning was then performed using the JASPAR database. Expression patterns of candidate TFs in pancreatic cancer and normal tissues were assessed using GEPIA. Based on UniProtKB/Swiss‐Prot annotation and expression context, ZNF263 was prioritized for downstream validation. ZNF263 ChIP‐seq datasets were surveyed in CistromeDB. Because ZNF263 ChIP‐seq data in pancreas or pancreatic cancer were not available in major public resources at the time of analysis, a representative high‐quality dataset from K562 cells was selected for track visualization.

### 4.11 Cell Lines

The human pancreatic cancer cell lines PANC‐1 (RRID: CVCL_0480), BxPC‐3 (RRID: CVCL_0186), and CFPAC‐1 (RRID: CVCL_1119) were purchased from Procell Life Science & Technology (Wuhan, China) in 2020. Cells were cultured in Dulbecco's Modified Eagle Medium (DMEM, Gibco, USA) supplemented with 10% fetal bovine serum (FBS, PAN, Germany), 100 U/mL penicillin, and 100 µg/mL streptomycin, and maintained at 37°C in a humidified incubator with 5% CO_2_. All cell lines were authenticated by short tandem repeat (STR) profiling (Genetic Testing Biotechnology Corporation, Suzhou, China) and confirmed to be free of mycoplasma contamination. No additional contamination was detected during the course of this study.

### 4.12 Dual‐Luciferase Reporter Assay

A 1000‐bp genomic DNA fragment encompassing either the *C* or *G* allele of rs11102484 was synthesized and cloned into the KpnI and XhoI sites of the pGL3‐Promoter vector (Promega, USA). The resulting reporter constructs were transfected into BxPC‐3 and PANC‐1 cells using Lipofectamine 3000 (Invitrogen, USA). After 36 h, cells were harvested and luciferase activity was measured using the Dual‐Luciferase Reporter Assay System (Promega, USA). Relative luciferase activity was calculated as the ratio of firefly to Renilla luciferase activity.

### 4.13 EMSA

Single‐stranded 25‐bp 3’ biotin‐labeled oligonucleotides containing either the *C* or *G* allele of rs11102484 were synthesized (TSINGKE Biological Technology, China) (Table S7) and annealed to form double‐stranded probes. Nuclear extracts were prepared from BxPC‐3 and PANC‐1 cells using the Nuclear and Cytoplasmic Protein Extraction Kit (Beyotime, China). EMSAs were performed using the Chemiluminescent EMSA Kit (Beyotime, China) according to the manufacturer's instructions.

### 4.14 ChIP‐qPCR

ChIP assays were conducted following the manufacturer's protocol of a ChIP kit (Millipore, USA). Briefly, cells were fixed with 1% formaldehyde, and cross‐linking was terminated by glycine treatment. The chromatin was then fragmented by sonication and immunoprecipitated overnight at 4°C using an anti‐ZNF263 antibody, with normal rabbit IgG serving as a negative control. The immunoprecipitated DNA was purified and subjected to qPCR analysis to determine the relative enrichment at specific genomic regions. The primers used for ChIP‐qPCR are shown in Table S7.

### 4.15 CRISPR‐dCas9‐KRAB Mediated Perturbation of Silencer

We designed site‐specific single guide RNAs (sgRNAs) (Table S7) for our candidate silencer by using publicly available filtering tools (http://crispor.gi.ucsc.edu/). For CRISPR interference, sgRNAs were cloned into the pLH‐spsgRNA2 (Addgene, #64114) through the BbsI site. Lentivirus was created by transfecting HEK293FT cells with sgRNA expression cocktails or pHAGE dCas9‐KRAB, together with Lenti‐Easy Packaging Mix (Genechem, #LPK001). After 8 h, cells were washed twice with PBS and fresh medium was added. Virus‐containing medium was collected 48–72 h after transfection, and filtered with 0.22 µm filters (Millipore). Stable cell lines were generated by infecting PDAC cell lines with lentivirus expressing dCas9‐KRAB and sgRNAs. Cells were then screened with puromycin (1 µg/mL, Amresco) for 48 h.

### 4.16 3C Assay

3C assays were conducted in pancreatic cancer cell lines carrying different genotypes of rs11102484, following a previously published protocol [48]. Briefly, cells were cross‐linked with formaldehyde, lysed, and digested overnight at 37°C with the restriction enzyme (New England Biolabs, USA). The chromatin fragments were then ligated at 16°C using T4 DNA ligase. DNA was purified by phenol‐chloroform extraction and ethanol precipitation. 3C interaction frequencies were quantified by qPCR, and a bacterial artificial chromosome (BAC) covering the target region was used to generate a control library for PCR efficiency normalization.

### 4.17 Expression Analysis of ST7L

*ST7L* expression across various cancer types was summarized using differential expression results from the Oncomine database comparing tumor and normal tissues. To assess *ST7L* expression across major cell types in pancreatic cancer, we analyzed an snRNA‐seq dataset, GSE202051 from GEO (18 tumor samples, including four with technical replicates), and an scRNA‐seq dataset, CRA001160 from the Genome Sequence Archive (GSA; 24 tumor samples). Processed gene expression matrices and cell‐type annotations were obtained from the original studies, and cells were grouped into major compartments. For each dataset, the percentage of *ST7L*‐positive cells and the scaled average expression (*z*‐score) were calculated for each cell type. In addition, curated pancreatic cancer single‐cell datasets (GSE111672, GSE148673, GSE154763, and GSE154778) were retrieved from the TISCH2 database, and cell type‐level *ST7L* expression values were summarized as a heatmap.

### 4.18 Plasmid Construction, Transfection and RT‐qPCR

The full‐length cDNA of *ST7L* was synthesized (Genewiz, China) and cloned into the NheI and KpnI sites of the pcDNA3.1(+) vector (Invitrogen, USA). Plasmids were then transfected into cells using Lipofectamine 3000 (Invitrogen, USA) for overexpression experiments. For gene knockdown, siRNAs targeting *ST7L* (Table S7) and non‐targeting control siRNAs were purchased from RiboBio (China). Transfections were performed using Lipofectamine RNAiMAX (Invitrogen, USA) at a final siRNA concentration of 100 nm. Total RNA was extracted using TRIzol reagent (Invitrogen, USA), and reverse transcription was carried out with PrimeScript RT Master Mix (Takara, Japan). RT‐qPCR was conducted with SYBR Green Master Mix (Takara, Japan) on a ViiA^TM^ 7 Real‐Time PCR System (Applied Biosystems, USA). *GAPDH* was used as the internal control, and relative mRNA levels were calculated using the 2^‐ΔΔCT^ method. Primers used in qPCR are shown in Table S7.

### 4.19 Cell Proliferation Assay

Cell proliferation was assessed using the CCK‐8 (Dojindo, Japan). BxPC‐3 and PANC‐1 cells were seeded into 96‐well plates at a density of 2 × 10^3^ cells per well. At designated time points, 10 µL of CCK‐8 reagent was added to each well and incubated at 37°C for 1.5 h. Absorbance at 450 nm was measured using a microplate reader (BioTek, USA).

### 4.20 Colony Formation Assay

After siRNA or plasmid transfection, BxPC‐3 and PANC‐1 cells were seeded into 6‐well plates at a density of 1000 cells per well. Cells were cultured for approximately two weeks with medium refreshed every 2–3 days. Colonies were fixed with 2 mL methanol for 30 min, stained with 0.25% crystal violet for 30 min, rinsed with deionized water, air‐dried, and imaged.

### 4.21 Western Blot

Total proteins were extracted from BxPC‐3 and PANC‐1 cell lines using RIPA buffer supplemented with protease and phosphatase inhibitors, followed by quantification and denaturation. After separation via 10% SDS‐PAGE, proteins were electrophoretically transferred to 0.45 µm PVDF membranes. The membranes were blocked and subsequently probed with the primary antibodies for ST7L (1:500, Proteintech, Cat No. 17567‐1‐AP), AKT (1:5000, Proteintech, Cat No. 60203‐2‐Ig), p‐AKT (1:2000, Proteintech, Cat No. 66444‐1‐Ig), β‐catenin (1:5000, Proteintech, Cat No. 51067‐2‐AP) and β‐Actin (1:20000, Proteintech, Cat No. 66009‐1‐Ig). HRP‐conjugated anti‐mouse IgG (1:5000, Proteintech, Cat No. SA00001‐1) or anti‐rabbit IgG (1:5000, Proteintech, Cat No. SA00001‐2) was used as the secondary antibody. Protein bands were finally detected using an ECL substrate.

### 4.22 Statistical Analyses

All statistical tests were two‐sided, and *p* < 0.05 was considered statistically significant. Data processing and normalization for high‐throughput analyses were detailed in the respective method subsections. For functional assays, data are presented as mean ± s.e.m. from at least three independent experiments, with replicate details provided in the figure legends. Between‐group comparisons used two‐tailed Student's *t*‐tests. For multiple testing, *P* values were adjusted by the BH method to control the FDR, unless stated otherwise. Effect sizes are reported with 95% CIs where applicable. Analyses were performed using R (v4.2.2) and GraphPad Prism (v10).

### 4.23 Ethics Approval Statement

The Clinical Research Institution Review Committee and Ethics Review Committee of Wuhan University (number: WHU‐LFMD‐IRB2023003) approved this study. All participants provided written informed consent.

## Author Contributions

X.W., H.G., Z.Y., Y.J., and C.C. conceived and designed the study. Z.L., S.T., M.Z., R.L., C.F., and B.L. provided technical support. X.W. and C.C. performed the analyses. X.W., H.G., Z.Y., and Y.J. contributed to the functional experiments and data interpretation. X.M., J.T., S.Z., and Y.Z. supervised the project and provided critical revisions. X.W. drafted the manuscript with input and edits from all co‐authors. All authors read and approved the final manuscript.

## Funding

This work was supported by National Key R&D Program of China (2024YFC3405804), Noncommunicable Chronic Diseases‐National Science and Technology Major Project (2023ZD0501400), National Natural Science Foundation of China (82373663, 82204128, 82322058, 82273713), Henan Provincial Medical Science and Technology Research Program—Provincial‐Ministerial Co‐constructed Youth Project (SBGJ202303015), Henan Provincial Science and Technology Research Program (242102310040), Natural Science Foundation of Hubei Province of China (2024AFB777) and Program of Health Commission of Hubei Province (WJ2023M045).

## Conflicts of Interest

The authors declare no conflicts of interest.

## Code Availability

The source code used in this study is available at https://github.com/XyWang1207/PancreaticCancer‐eQTL‐code.

## Supporting information




**Supporting file**: advs74809‐sup‐0001‐SuppMat.docx

## Data Availability

The PanC4 GWAS data used in this study are available from dbGaP (phs000648.v1.p1; https://dbgap.ncbi.nlm.nih.gov/beta/home). The eQTL data were obtained from the GTEx Project (v8; https://www.gtexportal.org/home/) and the TCGA Pancreatic Adenocarcinoma (PAAD) cohort (https://portal.gdc.cancer.gov/). All data and code supporting the findings of this study are available from the corresponding author upon reasonable request.
