# Supplementary material for: Mapping Genetic Regulation of Transcription to Identify Functional Variants and Genes Associated with Pancreatic Cancer Risk
Source: Adv Sci (Weinh). 2026 Mar 13;13(29):e17184. doi: 10.1002/advs.202517184 (PMC13205582; doi:10.1002/advs.202517184)
Supplement: Supplementary file 1 — Supporting file: advs74809‐sup‐0001‐SuppMat.docx [file ADVS-13-e17184-s001.docx]

Supporting Information

**Mapping Genetic Regulation of Transcription to Identify Functional Variants and Genes Associated with Pancreatic Cancer Risk**

*Xiaoyang Wang,* *Hui Geng, Zhengyan Yao, Yuan Jiang, Can Chen, Zequn Lu, Shuangshuang Tian, Ming Zhang, Ruiyan Liu, Chenxi Feng, Bin Li, Xiaoping Miao,* *Jianbo Tian*,* *Shaokai Zhang* and* *Ying Zhu**

**Supplementary tables**

**Table S1.** Characteristics of pancreatic cancer cases and controls in the GWAS meta-analysis.

| Variable | Chinese GWAS | |  | PanC4 GWAS | |  | Meta-analysis | |
| --- | --- | --- | --- | --- | --- | --- | --- | --- |
|  | Cases  n (%) | Controls  n (%) |  | Cases  n (%) | Controls  n (%) |  | Cases  n (%) | Controls  n (%) |
| Age, years^a)^ |  |  |  |  |  |  |  |  |
| < 60  ≥ 60 | 449 (45.77%) | 712 (35.76%) |  | 1,292 (31.16%) | 1,367 (36.14%) |  | 1,741 (33.96%) | 2,079 (36.01%) |
|  | 532 (54.23%) | 1,279 (64.24%) |  | 2,854 (68.84%) | 2,416 (63.86%) |  | 3,386 (66.04%) | 3,695 (63.99%) |
| Gender |  |  |  |  |  |  |  |  |
| Male  Female | 778 (79.31%) | 1,664 (83.58%) |  | 2,386 (57.51%) | 2,099 (55.46%) |  | 3,164 (61.68%) | 3,763 (65.15%) |
|  | 203 (20.69%) | 327 (16.42%) |  | 1,763 (42.49%) | 1,686 (44.54%) |  | 1,966 (38.32%) | 2,013 (34.85%) |
| Total | 981 | 1,991 |  | 4,149 | 3,785 |  | 5,130 | 5,776 |

^a)^Some age values were missing in the PanC4 GWAS.

**Table S2.** Pancreatic cancer risk loci identified in the GWAS meta-analysis.

| SNP^a)^ | Cytoband | RA | EA | OR (95% CI) | *P*_meta_ | *I*^2^ (%) | *P*_Het_ |
| --- | --- | --- | --- | --- | --- | --- | --- |
| rs351365 | 1p13.2 | G | A | 0.85 (0.80-0.90) | 3.91 × 10^-7^ | 0 | 0.703 |
| rs6702147 | 1p31.3 | G | A | 0.85 (0.79-0.91) | 8.86 × 10^-6^ | 0 | 0.905 |
| rs12747682 | 1p36.13 | A | G | 1.16 (1.09-1.23) | 3.65 × 10^-6^ | 0 | 0.990 |
| rs3790854 | 1q32.1 | A | G | 0.82 (0.77-0.87) | 1.03 × 10^-10^ | 0 | 0.371 |
| rs3770657 | 2p14 | A | G | 1.18 (1.11-1.25) | 2.49 × 10^-8^ | 68 | 0.077 |
| rs1992767 | 2p21 | T | C | 1.14 (1.08-1.21) | 5.55 × 10^-6^ | 0 | 0.730 |
| rs1515497 | 3q28 | T | C | 0.87 (0.82-0.92) | 4.62 × 10^-6^ | 0 | 0.511 |
| rs401681 | 5p15.33 | G | A | 1.20 (1.14-1.27) | 8.02 × 10^-11^ | 0 | 0.666 |
| rs347345 | 5q14.3 | C | T | 0.87 (0.82-0.92) | 1.92 × 10^-6^ | 22 | 0.258 |
| rs2325469 | 6q16.1 | C | T | 1.20 (1.11-1.29) | 1.61 × 10^-6^ | 0 | 0.703 |
| rs75907313 | 6q23.2 | T | C | 1.80 (1.52-2.14) | 1.82 × 10^-11^ | 0 | 0.352 |
| rs1733483 | 7p14.1 | A | G | 0.86 (0.81-0.91) | 6.79 × 10^-8^ | 0 | 0.919 |
| rs3779238 | 7p14.3 | C | T | 1.18 (1.10-1.27) | 9.80 × 10^-6^ | 0 | 0.913 |
| rs73291844 | 7p21.2 | G | T | 0.77 (0.68-0.86) | 5.08 × 10^-6^ | 0 | 0.776 |
| rs7016016 | 8q13.1 | A | G | 1.25 (1.13-1.37) | 8.07 × 10^-6^ | 0 | 0.494 |
| rs498010 | 9p23 | G | A | 1.17 (1.10-1.26) | 4.07 × 10^-6^ | 51 | 0.152 |
| rs630103 | 9q31.1 | G | A | 1.18 (1.11-1.24) | 8.07 × 10^-9^ | 0 | 0.972 |
| rs9411378 | 9q34.2 | C | A | 1.25 (1.18-1.33) | 4.43 × 10^-14^ | 0 | 0.336 |
| rs76059813 | 10p11.21 | C | T | 0.78 (0.70-0.86) | 2.35 × 10^-6^ | 51 | 0.154 |
| rs2796326 | 10p12.1 | G | A | 0.88 (0.83-0.93) | 5.43 × 10^-6^ | 20 | 0.265 |
| rs10898207 | 11q14.1 | T | A | 1.22 (1.12-1.32) | 4.10 × 10^-6^ | 0 | 0.840 |
| rs11618581 | 13q12.2 | G | A | 1.19 (1.13-1.26) | 7.73 × 10^-10^ | 0 | 0.416 |
| rs596474 | 13q13.1 | A | G | 0.87 (0.82-0.92) | 6.45 × 10^-7^ | 33 | 0.223 |
| rs1945514 | 13q13.3 | A | T | 1.15 (1.08-1.22) | 3.40 × 10^-6^ | 0 | 0.956 |
| rs1411328 | 13q22.1 | G | A | 1.17 (1.11-1.24) | 4.38 × 10^-8^ | 0 | 0.443 |
| rs7148132 | 14q23.1 | A | G | 1.15 (1.09-1.22) | 1.03 × 10^-6^ | 0 | 0.486 |
| rs74073707 | 14q32.12 | T | C | 1.25 (1.14-1.37) | 2.96 × 10^-6^ | 0 | 0.709 |
| rs2735817 | 14q32.33 | G | A | 1.18 (1.11-1.26) | 4.88 × 10^-7^ | 48 | 0.164 |
| rs10468160 | 15q11.2 | G | C | 0.84 (0.78-0.90) | 3.79 × 10^-6^ | 20 | 0.263 |
| rs78855037 | 15q21.3 | T | A | 1.30 (1.17-1.44) | 1.17 × 10^-6^ | 0 | 0.669 |
| rs11646044 | 16q23.1 | G | T | 1.19 (1.11-1.27) | 8.64 × 10^-7^ | 0 | 0.888 |
| rs407307 | 17q12 | A | G | 1.15 (1.09-1.23) | 3.14 × 10^-6^ | 0 | 0.658 |
| rs11655237 | 17q24.3 | C | T | 1.35 (1.25-1.45) | 2.66 × 10^-14^ | 0 | 0.398 |
| rs640431 | 18q12.3 | G | T | 0.86 (0.81-0.91) | 2.41 × 10^-6^ | 0 | 0.430 |
| rs2104351 | 20p12.1 | A | C | 0.85 (0.80-0.90) | 4.83 × 10^-7^ | 0 | 0.676 |

RA, reference allele; EA, effect Allele; OR, odds ratio; CI, confidence interval; *P*_meta_, *P* value from the GWAS meta-analysis; *I*^2^, percentage of total variation across studies due to heterogeneity; *P*_Het_, *P* value for heterogeneity (Cochran’s Q test).

^a)^The most significant independent signal at each locus.

**Table S3.** Genomic annotation of candidate functional genes prioritized by the integrative analysis.

| Gene ID | Gene symbol | Chromosome | Gene start (bp)^a)^ | Gene end (bp)^a)^ | Strand^b)^ | Gene type |
| --- | --- | --- | --- | --- | --- | --- |
| ENSG00000127463 | *EMC1* | 1 | 19542158 | 19578046 | - | Protein-coding gene |
| ENSG00000007341 | *ST7L* | 1 | 113066140 | 113163447 | - | Protein-coding gene |
| ENSG00000143971 | *ETAA1* | 2 | 67624451 | 67637677 | + | Protein-coding gene |
| ENSG00000122641 | *INHBA* | 7 | 41724712 | 41742706 | - | Protein-coding gene |
| ENSG00000139515 | *PDX1* | 13 | 28494157 | 28500368 | + | Protein-coding gene |
| ENSG00000279314 | NA | 13 | 32534462 | 32536083 | - | TEC |
| ENSG00000185024 | *BRF1* | 14 | 105675623 | 105781926 | - | Protein-coding gene |
| ENSG00000184887 | *BTBD6* | 14 | 105714827 | 105717430 | + | Protein-coding gene |
| ENSG00000211896 | *IGHG1* | 14 | 106202680 | 106209408 | - | IG C gene |
| ENSG00000090863 | *GLG1* | 16 | 74485856 | 74641012 | - | Protein-coding gene |
| ENSG00000240338 | NA | 16 | 75259972 | 75262093 | + | Pseudogene |
| ENSG00000153774 | *CFDP1* | 16 | 75327596 | 75467383 | - | Protein-coding gene |
| ENSG00000261783 | NA | 16 | 75413716 | 75415158 | - | Long non-coding RNA gene |
| ENSG00000161395 | *PGAP3* | 17 | 37827375 | 37853050 | - | Protein-coding gene |
| ENSG00000073605 | *GSDMB* | 17 | 38060848 | 38076107 | - | Protein-coding gene |

IG C gene, constant chain immunoglobulin gene undergoing somatic recombination before transcription; TEC, to be experimentally confirmed; NA, not available.

^a)^Position of gene according to NCBI Human Genome Build 37 (hg19).

^b)^+, forward; -, reverse.

**Table S4.** Summary of eQTL meta-analysis results and heterogeneity metrics for GWAS-eQTL integrated SNP-gene pairs.

| SNP | CHR | BP | Alleles | Gene | *β*_GTEx_ | *P*_GTEx_ | *β*_TCGA_ | *P*_TCGA_ | *β*_meta_ | *P*_meta_ | *P*_Het_ | *I*^2^ |
| --- | --- | --- | --- | --- | --- | --- | --- | --- | --- | --- | --- | --- |
| rs11102484 | 1 | 113051730 | C/G | *ST7L* | 0.230 | 1.87E-03 | 0.278 | 6.20E-04 | 0.252 | 4.16E-06 | 0.660 | 0 |
| rs3828074 | 1 | 113050985 | C/T | *ST7L* | 0.230 | 1.87E-03 | 0.284 | 5.33E-04 | 0.254 | 3.68E-06 | 0.623 | 0 |
| rs12136566 | 1 | 113051074 | A/G | *ST7L* | 0.230 | 1.87E-03 | 0.284 | 5.16E-04 | 0.254 | 3.58E-06 | 0.620 | 0 |
| rs3790605 | 1 | 113052155 | C/G | *ST7L* | 0.230 | 1.87E-03 | 0.269 | 8.69E-04 | 0.247 | 5.58E-06 | 0.721 | 0 |
| rs12747682 | 1 | 19354505 | A/G | *EMC1* | -0.287 | 1.98E-08 | -0.058 | 2.25E-01 | -0.165 | 2.31E-06 | 0.001 | 91 |
| rs1483351 | 2 | 67520575 | G/C | *ETAA1* | -0.180 | 3.78E-03 | -0.212 | 2.08E-03 | -0.194 | 2.52E-05 | 0.732 | 0 |
| rs7564335 | 2 | 67579967 | A/G | *ETAA1* | -0.148 | 1.79E-02 | -0.229 | 1.55E-03 | -0.183 | 1.12E-04 | 0.399 | 0 |
| rs6546298 | 2 | 67581853 | T/G | *ETAA1* | -0.148 | 1.79E-02 | -0.229 | 1.55E-03 | -0.183 | 1.12E-04 | 0.399 | 0 |
| rs6739471 | 2 | 67585593 | T/C | *ETAA1* | -0.148 | 1.79E-02 | -0.231 | 1.43E-03 | -0.184 | 1.06E-04 | 0.390 | 0 |
| rs11890682 | 2 | 67589585 | A/G | *ETAA1* | -0.148 | 1.79E-02 | -0.231 | 1.43E-03 | -0.184 | 1.06E-04 | 0.390 | 0 |
| rs1601530 | 2 | 67594916 | G/A | *ETAA1* | -0.139 | 2.62E-02 | -0.231 | 1.43E-03 | -0.179 | 1.65E-04 | 0.340 | 0 |
| rs6546300 | 2 | 67595196 | T/G | *ETAA1* | -0.148 | 1.79E-02 | -0.231 | 1.43E-03 | -0.184 | 1.06E-04 | 0.390 | 0 |
| rs6714988 | 2 | 67595789 | A/G | *ETAA1* | -0.139 | 2.62E-02 | -0.231 | 1.43E-03 | -0.179 | 1.65E-04 | 0.340 | 0 |
| rs1027000 | 2 | 67600789 | T/C | *ETAA1* | -0.139 | 2.62E-02 | -0.230 | 1.53E-03 | -0.178 | 1.75E-04 | 0.344 | 0 |
| rs1032158 | 2 | 67601028 | A/C | *ETAA1* | -0.139 | 2.62E-02 | -0.230 | 1.53E-03 | -0.178 | 1.75E-04 | 0.344 | 0 |
| rs6732641 | 2 | 67608585 | T/C | *ETAA1* | -0.139 | 2.62E-02 | -0.231 | 1.43E-03 | -0.179 | 1.65E-04 | 0.340 | 0 |
| rs3770657 | 2 | 67630980 | G/A | *ETAA1* | -0.127 | 3.82E-02 | -0.240 | 9.30E-04 | -0.174 | 1.98E-04 | 0.235 | 29 |
| rs13246232 | 7 | 40871858 | A/G | *INHBA* | 0.221 | 1.42E-05 | -0.002 | 9.79E-01 | 0.131 | 8.59E-04 | 0.005 | 87 |
| rs1733483 | 7 | 40873221 | A/G | *INHBA* | 0.219 | 2.00E-05 | 0.028 | 6.11E-01 | 0.128 | 5.78E-04 | 0.010 | 85 |
| rs11618581 | 13 | 28476911 | G/A | *PDX1* | -0.281 | 3.74E-07 | -0.038 | 5.32E-01 | -0.169 | 3.20E-05 | 0.003 | 89 |
| rs9554197 | 13 | 28476978 | C/T | *PDX1* | -0.281 | 3.74E-07 | -0.038 | 5.32E-01 | -0.169 | 3.20E-05 | 0.003 | 89 |
| rs594422 | 13 | 32455797 | A/G | *ENSG00000279314* | -0.151 | 1.60E-02 | -0.230 | 7.17E-03 | -0.179 | 4.10E-04 | 0.458 | 0 |
| rs606589 | 13 | 32456150 | T/C | *ENSG00000279314* | -0.152 | 1.51E-02 | -0.230 | 7.17E-03 | -0.179 | 3.87E-04 | 0.463 | 0 |
| rs595971 | 13 | 32457157 | C/T | *ENSG00000279314* | -0.147 | 1.89E-02 | -0.230 | 7.17E-03 | -0.176 | 4.97E-04 | 0.432 | 0 |
| rs596375 | 13 | 32457197 | A/G | *ENSG00000279314* | -0.147 | 1.89E-02 | -0.230 | 7.17E-03 | -0.176 | 4.97E-04 | 0.432 | 0 |
| rs596474 | 13 | 32457262 | G/A | *ENSG00000279314* | -0.158 | 1.19E-02 | -0.230 | 7.17E-03 | -0.183 | 2.98E-04 | 0.496 | 0 |
| rs622444 | 13 | 32457425 | T/C | *ENSG00000279314* | -0.143 | 2.22E-02 | -0.230 | 7.17E-03 | -0.174 | 5.93E-04 | 0.414 | 0 |
| rs598637 | 13 | 32457690 | A/G | *ENSG00000279314* | -0.143 | 2.22E-02 | -0.230 | 7.17E-03 | -0.174 | 5.93E-04 | 0.414 | 0 |
| rs612045 | 13 | 32458430 | A/G | *ENSG00000279314* | -0.152 | 1.51E-02 | -0.230 | 7.17E-03 | -0.179 | 3.87E-04 | 0.463 | 0 |
| rs670502 | 13 | 32462277 | G/C | *ENSG00000279314* | -0.142 | 2.36E-02 | -0.231 | 7.00E-03 | -0.173 | 6.25E-04 | 0.404 | 0 |
| rs203424 | 13 | 32467899 | C/A | *ENSG00000279314* | -0.147 | 1.89E-02 | -0.231 | 7.00E-03 | -0.176 | 4.88E-04 | 0.429 | 0 |
| rs23506 | 13 | 32468990 | A/T | *ENSG00000279314* | -0.142 | 2.63E-02 | -0.231 | 7.00E-03 | -0.173 | 6.90E-04 | 0.405 | 0 |
| rs619396 | 13 | 32472273 | T/C | *ENSG00000279314* | -0.148 | 1.82E-02 | -0.231 | 7.00E-03 | -0.177 | 4.65E-04 | 0.439 | 0 |
| rs7146643 | 14 | 105757392 | T/C | *BTBD6* | 0.280 | 1.43E-06 | 0.024 | 5.86E-01 | 0.119 | 7.71E-04 | 0.000 | 92 |
| rs3000075 | 14 | 105713219 | A/G | *BRF1* | 0.163 | 2.05E-05 | 0.022 | 6.52E-01 | 0.110 | 2.69E-04 | 0.024 | 80 |
| rs2735817 | 14 | 105717796 | G/A | *BRF1* | 0.168 | 2.16E-05 | 0.019 | 6.98E-01 | 0.110 | 3.70E-04 | 0.019 | 82 |
| rs2816608 | 14 | 105718407 | C/T | *BRF1* | 0.151 | 4.15E-05 | 0.030 | 5.30E-01 | 0.106 | 2.85E-04 | 0.045 | 75 |
| rs2816632 | 14 | 105741355 | T/C | *BRF1* | 0.146 | 7.06E-05 | 0.030 | 5.30E-01 | 0.103 | 4.14E-04 | 0.054 | 73 |
| rs2735817 | 14 | 105717796 | G/A | *IGHG1* | -0.189 | 1.00E-02 | -0.173 | 3.29E-02 | -0.182 | 8.39E-04 | 0.884 | 0 |
| rs4594277 | 16 | 75314023 | G/C | *GLG1* | -0.117 | 1.20E-03 | -0.065 | 2.58E-01 | -0.102 | 8.22E-04 | 0.450 | 0 |
| rs2161648 | 16 | 75314629 | T/G | *GLG1* | -0.119 | 1.07E-03 | -0.064 | 2.61E-01 | -0.103 | 7.78E-04 | 0.415 | 0 |
| rs4888418 | 16 | 75460306 | C/G | *GLG1* | -0.107 | 3.22E-03 | -0.119 | 3.49E-02 | -0.110 | 2.96E-04 | 0.862 | 0 |
| rs11862684 | 16 | 75461746 | T/G | *GLG1* | -0.106 | 3.47E-03 | -0.120 | 3.33E-02 | -0.110 | 3.06E-04 | 0.840 | 0 |
| rs11861810 | 16 | 75461829 | A/G | *GLG1* | -0.106 | 3.47E-03 | -0.119 | 3.48E-02 | -0.110 | 3.17E-04 | 0.850 | 0 |
| rs12930452 | 16 | 75462055 | A/G | *GLG1* | -0.106 | 3.47E-03 | -0.118 | 3.53E-02 | -0.110 | 3.21E-04 | 0.854 | 0 |
| rs12929908 | 16 | 75462071 | C/T | *GLG1* | -0.106 | 3.47E-03 | -0.117 | 3.69E-02 | -0.109 | 3.34E-04 | 0.865 | 0 |
| rs12928898 | 16 | 75462081 | G/A | *GLG1* | -0.106 | 3.47E-03 | -0.117 | 3.69E-02 | -0.109 | 3.34E-04 | 0.865 | 0 |
| rs11149832 | 16 | 75462713 | C/T | *GLG1* | -0.106 | 3.47E-03 | -0.119 | 3.48E-02 | -0.110 | 3.17E-04 | 0.850 | 0 |
| rs12448947 | 16 | 75462954 | G/A | *GLG1* | -0.106 | 3.47E-03 | -0.119 | 3.48E-02 | -0.110 | 3.17E-04 | 0.850 | 0 |
| rs4888420 | 16 | 75462998 | G/C | *GLG1* | -0.106 | 3.47E-03 | -0.124 | 2.95E-02 | -0.111 | 2.77E-04 | 0.795 | 0 |
| rs12449170 | 16 | 75463012 | C/T | *GLG1* | -0.106 | 3.47E-03 | -0.124 | 2.95E-02 | -0.111 | 2.77E-04 | 0.795 | 0 |
| rs11149833 | 16 | 75464002 | G/C | *GLG1* | -0.106 | 3.47E-03 | -0.119 | 3.48E-02 | -0.110 | 3.17E-04 | 0.850 | 0 |
| rs11641587 | 16 | 75464419 | G/T | *GLG1* | -0.106 | 3.47E-03 | -0.119 | 3.48E-02 | -0.110 | 3.17E-04 | 0.850 | 0 |
| rs12930768 | 16 | 75464500 | T/C | *GLG1* | -0.106 | 3.47E-03 | -0.119 | 3.48E-02 | -0.110 | 3.17E-04 | 0.850 | 0 |
| rs12445726 | 16 | 75465575 | C/T | *GLG1* | -0.106 | 3.47E-03 | -0.119 | 3.48E-02 | -0.110 | 3.17E-04 | 0.850 | 0 |
| rs11644741 | 16 | 75466433 | C/A | *GLG1* | -0.110 | 2.30E-03 | -0.115 | 3.98E-02 | -0.111 | 2.36E-04 | 0.943 | 0 |
| rs12917651 | 16 | 75466696 | G/T | *GLG1* | -0.106 | 3.47E-03 | -0.115 | 3.98E-02 | -0.109 | 3.55E-04 | 0.898 | 0 |
| rs35787595 | 16 | 75466847 | G/C | *GLG1* | -0.106 | 3.47E-03 | -0.116 | 3.80E-02 | -0.109 | 3.42E-04 | 0.886 | 0 |
| rs12929673 | 16 | 75470295 | C/T | *GLG1* | -0.110 | 2.30E-03 | -0.118 | 3.44E-02 | -0.112 | 2.08E-04 | 0.907 | 0 |
| rs35209155 | 16 | 75470496 | G/A | *GLG1* | -0.110 | 2.30E-03 | -0.118 | 3.44E-02 | -0.112 | 2.08E-04 | 0.907 | 0 |
| rs8055974 | 16 | 75470986 | C/G | *GLG1* | -0.110 | 2.17E-03 | -0.118 | 3.44E-02 | -0.112 | 1.97E-04 | 0.909 | 0 |
| rs4887825 | 16 | 75472422 | C/A | *GLG1* | -0.110 | 2.17E-03 | -0.119 | 3.31E-02 | -0.113 | 1.91E-04 | 0.898 | 0 |
| rs34021527 | 16 | 75474090 | A/G | *GLG1* | -0.107 | 3.04E-03 | -0.119 | 3.32E-02 | -0.111 | 2.67E-04 | 0.866 | 0 |
| rs56004344 | 16 | 75474189 | T/C | *GLG1* | -0.107 | 3.04E-03 | -0.119 | 3.32E-02 | -0.111 | 2.67E-04 | 0.866 | 0 |
| rs7204984 | 16 | 75475520 | G/C | *GLG1* | -0.108 | 2.92E-03 | -0.113 | 4.38E-02 | -0.109 | 3.26E-04 | 0.933 | 0 |
| rs35214308 | 16 | 75476088 | C/T | *GLG1* | -0.111 | 1.93E-03 | -0.113 | 4.38E-02 | -0.112 | 2.17E-04 | 0.976 | 0 |
| rs4888425 | 16 | 75476363 | T/C | *GLG1* | -0.108 | 2.92E-03 | -0.113 | 4.38E-02 | -0.109 | 3.26E-04 | 0.933 | 0 |
| rs4888426 | 16 | 75476451 | G/A | *GLG1* | -0.108 | 2.92E-03 | -0.113 | 4.38E-02 | -0.109 | 3.26E-04 | 0.933 | 0 |
| rs2161684 | 16 | 75479153 | T/C | *GLG1* | -0.108 | 2.92E-03 | -0.106 | 6.11E-02 | -0.107 | 4.38E-04 | 0.983 | 0 |
| rs35683383 | 16 | 75479277 | G/T | *GLG1* | -0.110 | 2.42E-03 | -0.088 | 1.18E-01 | -0.103 | 6.81E-04 | 0.746 | 0 |
| rs34996006 | 16 | 75479434 | A/G | *GLG1* | -0.108 | 2.92E-03 | -0.106 | 6.11E-02 | -0.107 | 4.38E-04 | 0.983 | 0 |
| rs12927562 | 16 | 75482860 | G/A | *GLG1* | -0.111 | 1.93E-03 | -0.106 | 6.11E-02 | -0.110 | 2.93E-04 | 0.939 | 0 |
| rs4887813 | 16 | 75312494 | A/G | *ENSG00000240338* | 0.176 | 9.66E-04 | 0.190 | 3.30E-02 | 0.180 | 8.61E-05 | 0.894 | 0 |
| rs6624 | 16 | 75327916 | A/G | *ENSG00000240338* | 0.188 | 4.05E-04 | 0.196 | 2.28E-02 | 0.190 | 2.60E-05 | 0.933 | 0 |
| rs6564249 | 16 | 75355756 | T/C | *ENSG00000240338* | 0.197 | 4.82E-04 | 0.249 | 5.32E-03 | 0.212 | 8.98E-06 | 0.627 | 0 |
| rs7199680 | 16 | 75355957 | T/C | *ENSG00000240338* | 0.156 | 1.48E-03 | 0.227 | 3.87E-03 | 0.176 | 2.38E-05 | 0.442 | 0 |
| rs9934007 | 16 | 75390532 | T/C | *ENSG00000240338* | 0.138 | 4.71E-03 | 0.182 | 1.84E-02 | 0.150 | 2.64E-04 | 0.630 | 0 |
| rs4887813 | 16 | 75312494 | A/G | *ENSG00000261783* | -0.235 | 2.17E-02 | -0.554 | 2.25E-05 | -0.356 | 9.85E-06 | 0.055 | 73 |
| rs11646044 | 16 | 75312548 | G/T | *ENSG00000261783* | -0.679 | 3.45E-17 | -0.712 | 3.28E-13 | -0.693 | 8.26E-29 | 0.797 | 0 |
| rs4888372 | 16 | 75313485 | G/A | *ENSG00000261783* | -0.695 | 4.84E-18 | -0.687 | 1.23E-12 | -0.692 | 4.20E-29 | 0.949 | 0 |
| rs4594277 | 16 | 75314023 | G/C | *ENSG00000261783* | -0.679 | 1.01E-17 | -0.712 | 3.28E-13 | -0.692 | 2.44E-29 | 0.792 | 0 |
| rs2161648 | 16 | 75314629 | T/G | *ENSG00000261783* | -0.685 | 1.51E-17 | -0.715 | 1.20E-13 | -0.697 | 1.34E-29 | 0.808 | 0 |
| rs1011121 | 16 | 75325933 | A/G | *ENSG00000261783* | -0.678 | 3.83E-17 | -0.707 | 5.24E-13 | -0.690 | 1.45E-28 | 0.817 | 0 |
| rs6624 | 16 | 75327916 | A/G | *ENSG00000261783* | -0.206 | 4.39E-02 | -0.514 | 5.17E-05 | -0.327 | 4.00E-05 | 0.059 | 72 |
| rs6564249 | 16 | 75355756 | T/C | *ENSG00000261783* | -0.228 | 3.59E-02 | -0.610 | 3.10E-06 | -0.384 | 4.33E-06 | 0.025 | 80 |
| rs7194129 | 16 | 75355977 | C/T | *ENSG00000261783* | -0.703 | 2.45E-18 | -0.752 | 6.62E-15 | -0.723 | 1.30E-31 | 0.699 | 0 |
| rs4888418 | 16 | 75460306 | C/G | *ENSG00000261783* | -0.713 | 1.84E-19 | -0.749 | 1.95E-15 | -0.728 | 2.86E-33 | 0.772 | 0 |
| rs11862684 | 16 | 75461746 | T/G | *ENSG00000261783* | -0.705 | 5.33E-19 | -0.747 | 2.20E-15 | -0.722 | 9.46E-33 | 0.728 | 0 |
| rs11861810 | 16 | 75461829 | A/G | *ENSG00000261783* | -0.705 | 5.33E-19 | -0.749 | 2.00E-15 | -0.723 | 8.60E-33 | 0.720 | 0 |
| rs12930452 | 16 | 75462055 | A/G | *ENSG00000261783* | -0.705 | 5.33E-19 | -0.748 | 2.21E-15 | -0.722 | 9.48E-33 | 0.726 | 0 |
| rs12929908 | 16 | 75462071 | C/T | *ENSG00000261783* | -0.705 | 5.33E-19 | -0.749 | 2.00E-15 | -0.723 | 8.62E-33 | 0.718 | 0 |
| rs12928898 | 16 | 75462081 | G/A | *ENSG00000261783* | -0.705 | 5.33E-19 | -0.749 | 2.00E-15 | -0.723 | 8.62E-33 | 0.718 | 0 |
| rs11149832 | 16 | 75462713 | C/T | *ENSG00000261783* | -0.705 | 5.33E-19 | -0.749 | 2.00E-15 | -0.723 | 8.60E-33 | 0.720 | 0 |
| rs12448947 | 16 | 75462954 | G/A | *ENSG00000261783* | -0.705 | 5.33E-19 | -0.749 | 2.00E-15 | -0.723 | 8.60E-33 | 0.720 | 0 |
| rs4888420 | 16 | 75462998 | G/C | *ENSG00000261783* | -0.705 | 5.33E-19 | -0.755 | 2.32E-15 | -0.725 | 1.02E-32 | 0.684 | 0 |
| rs12449170 | 16 | 75463012 | C/T | *ENSG00000261783* | -0.705 | 5.33E-19 | -0.755 | 2.32E-15 | -0.725 | 1.02E-32 | 0.684 | 0 |
| rs11149833 | 16 | 75464002 | G/C | *ENSG00000261783* | -0.705 | 5.33E-19 | -0.749 | 2.00E-15 | -0.723 | 8.60E-33 | 0.720 | 0 |
| rs11641587 | 16 | 75464419 | G/T | *ENSG00000261783* | -0.705 | 5.33E-19 | -0.749 | 2.00E-15 | -0.723 | 8.60E-33 | 0.720 | 0 |
| rs12930768 | 16 | 75464500 | T/C | *ENSG00000261783* | -0.705 | 5.33E-19 | -0.749 | 2.00E-15 | -0.723 | 8.60E-33 | 0.720 | 0 |
| rs12445726 | 16 | 75465575 | C/T | *ENSG00000261783* | -0.705 | 5.33E-19 | -0.749 | 2.00E-15 | -0.723 | 8.60E-33 | 0.720 | 0 |
| rs11644741 | 16 | 75466433 | C/A | *ENSG00000261783* | -0.696 | 8.96E-19 | -0.749 | 9.90E-16 | -0.718 | 7.41E-33 | 0.667 | 0 |
| rs12917651 | 16 | 75466696 | G/T | *ENSG00000261783* | -0.705 | 5.33E-19 | -0.749 | 9.90E-16 | -0.723 | 4.30E-33 | 0.718 | 0 |
| rs35787595 | 16 | 75466847 | G/C | *ENSG00000261783* | -0.705 | 5.33E-19 | -0.747 | 1.09E-15 | -0.723 | 4.72E-33 | 0.726 | 0 |
| rs12929673 | 16 | 75470295 | C/T | *ENSG00000261783* | -0.696 | 8.96E-19 | -0.735 | 3.64E-15 | -0.712 | 2.58E-32 | 0.752 | 0 |
| rs35209155 | 16 | 75470496 | G/A | *ENSG00000261783* | -0.696 | 8.96E-19 | -0.735 | 3.64E-15 | -0.712 | 2.58E-32 | 0.752 | 0 |
| rs8055974 | 16 | 75470986 | C/G | *ENSG00000261783* | -0.689 | 1.78E-18 | -0.735 | 3.64E-15 | -0.708 | 5.22E-32 | 0.704 | 0 |
| rs4887825 | 16 | 75472422 | C/A | *ENSG00000261783* | -0.689 | 1.78E-18 | -0.735 | 3.66E-15 | -0.708 | 5.25E-32 | 0.703 | 0 |
| rs34021527 | 16 | 75474090 | A/G | *ENSG00000261783* | -0.706 | 3.68E-19 | -0.736 | 3.50E-15 | -0.718 | 1.01E-32 | 0.806 | 0 |
| rs56004344 | 16 | 75474189 | T/C | *ENSG00000261783* | -0.706 | 3.68E-19 | -0.736 | 3.50E-15 | -0.718 | 1.01E-32 | 0.806 | 0 |
| rs7204984 | 16 | 75475520 | G/C | *ENSG00000261783* | -0.704 | 3.76E-19 | -0.724 | 2.06E-14 | -0.712 | 5.85E-32 | 0.871 | 0 |
| rs35214308 | 16 | 75476088 | C/T | *ENSG00000261783* | -0.696 | 6.49E-19 | -0.724 | 2.06E-14 | -0.707 | 1.02E-31 | 0.816 | 0 |
| rs4888425 | 16 | 75476363 | T/C | *ENSG00000261783* | -0.704 | 3.76E-19 | -0.724 | 2.06E-14 | -0.712 | 5.85E-32 | 0.871 | 0 |
| rs4888426 | 16 | 75476451 | G/A | *ENSG00000261783* | -0.704 | 3.76E-19 | -0.724 | 2.06E-14 | -0.712 | 5.85E-32 | 0.871 | 0 |
| rs2161684 | 16 | 75479153 | T/C | *ENSG00000261783* | -0.704 | 3.76E-19 | -0.738 | 9.59E-15 | -0.718 | 2.80E-32 | 0.786 | 0 |
| rs35683383 | 16 | 75479277 | G/T | *ENSG00000261783* | -0.704 | 4.53E-19 | -0.728 | 1.52E-14 | -0.714 | 5.22E-32 | 0.847 | 0 |
| rs34996006 | 16 | 75479434 | A/G | *ENSG00000261783* | -0.704 | 3.76E-19 | -0.738 | 9.59E-15 | -0.718 | 2.80E-32 | 0.786 | 0 |
| rs12927562 | 16 | 75482860 | G/A | *ENSG00000261783* | -0.696 | 6.49E-19 | -0.738 | 9.59E-15 | -0.712 | 4.93E-32 | 0.733 | 0 |
| rs4887813 | 16 | 75312494 | A/G | *CFDP1* | -0.172 | 3.91E-04 | -0.138 | 9.81E-02 | -0.164 | 9.75E-05 | 0.722 | 0 |
| rs11646044 | 16 | 75312548 | G/T | *CFDP1* | -0.118 | 4.09E-03 | -0.159 | 1.56E-02 | -0.129 | 2.02E-04 | 0.595 | 0 |
| rs4888372 | 16 | 75313485 | G/A | *CFDP1* | -0.115 | 5.09E-03 | -0.134 | 3.92E-02 | -0.120 | 5.21E-04 | 0.806 | 0 |
| rs4594277 | 16 | 75314023 | G/C | *CFDP1* | -0.120 | 2.85E-03 | -0.159 | 1.56E-02 | -0.131 | 1.40E-04 | 0.618 | 0 |
| rs2161648 | 16 | 75314629 | T/G | *CFDP1* | -0.127 | 1.81E-03 | -0.159 | 1.44E-02 | -0.136 | 8.03E-05 | 0.682 | 0 |
| rs1011121 | 16 | 75325933 | A/G | *CFDP1* | -0.118 | 3.98E-03 | -0.168 | 1.05E-02 | -0.132 | 1.46E-04 | 0.518 | 0 |
| rs6624 | 16 | 75327916 | A/G | *CFDP1* | -0.171 | 4.35E-04 | -0.151 | 6.18E-02 | -0.165 | 6.98E-05 | 0.834 | 0 |
| rs6564249 | 16 | 75355756 | T/C | *CFDP1* | -0.177 | 6.00E-04 | -0.124 | 1.41E-01 | -0.163 | 2.21E-04 | 0.588 | 0 |
| rs7199680 | 16 | 75355957 | T/C | *CFDP1* | -0.228 | 2.05E-07 | -0.150 | 4.30E-02 | -0.208 | 3.81E-08 | 0.359 | 0 |
| rs7194129 | 16 | 75355977 | C/T | *CFDP1* | -0.110 | 7.77E-03 | -0.131 | 4.68E-02 | -0.116 | 9.29E-04 | 0.786 | 0 |
| rs9934007 | 16 | 75390532 | T/C | *CFDP1* | -0.216 | 8.04E-07 | -0.156 | 3.06E-02 | -0.200 | 9.30E-08 | 0.476 | 0 |
| rs4888418 | 16 | 75460306 | C/G | *CFDP1* | -0.116 | 4.39E-03 | -0.173 | 7.04E-03 | -0.132 | 1.19E-04 | 0.452 | 0 |
| rs11862684 | 16 | 75461746 | T/G | *CFDP1* | -0.118 | 3.59E-03 | -0.173 | 6.82E-03 | -0.134 | 9.33E-05 | 0.466 | 0 |
| rs11861810 | 16 | 75461829 | A/G | *CFDP1* | -0.118 | 3.59E-03 | -0.173 | 6.90E-03 | -0.134 | 9.42E-05 | 0.467 | 0 |
| rs12930452 | 16 | 75462055 | A/G | *CFDP1* | -0.118 | 3.59E-03 | -0.174 | 6.82E-03 | -0.134 | 9.34E-05 | 0.465 | 0 |
| rs12929908 | 16 | 75462071 | C/T | *CFDP1* | -0.118 | 3.59E-03 | -0.173 | 6.90E-03 | -0.134 | 9.43E-05 | 0.467 | 0 |
| rs12928898 | 16 | 75462081 | G/A | *CFDP1* | -0.118 | 3.59E-03 | -0.173 | 6.90E-03 | -0.134 | 9.43E-05 | 0.467 | 0 |
| rs11149832 | 16 | 75462713 | C/T | *CFDP1* | -0.118 | 3.59E-03 | -0.173 | 6.90E-03 | -0.134 | 9.42E-05 | 0.467 | 0 |
| rs12448947 | 16 | 75462954 | G/A | *CFDP1* | -0.118 | 3.59E-03 | -0.173 | 6.90E-03 | -0.134 | 9.42E-05 | 0.467 | 0 |
| rs4888420 | 16 | 75462998 | G/C | *CFDP1* | -0.118 | 3.59E-03 | -0.166 | 1.03E-02 | -0.132 | 1.29E-04 | 0.528 | 0 |
| rs12449170 | 16 | 75463012 | C/T | *CFDP1* | -0.118 | 3.59E-03 | -0.166 | 1.03E-02 | -0.132 | 1.29E-04 | 0.528 | 0 |
| rs11149833 | 16 | 75464002 | G/C | *CFDP1* | -0.118 | 3.59E-03 | -0.173 | 6.90E-03 | -0.134 | 9.42E-05 | 0.467 | 0 |
| rs11641587 | 16 | 75464419 | G/T | *CFDP1* | -0.118 | 3.59E-03 | -0.173 | 6.90E-03 | -0.134 | 9.42E-05 | 0.467 | 0 |
| rs12930768 | 16 | 75464500 | T/C | *CFDP1* | -0.118 | 3.59E-03 | -0.173 | 6.90E-03 | -0.134 | 9.42E-05 | 0.467 | 0 |
| rs12445726 | 16 | 75465575 | C/T | *CFDP1* | -0.118 | 3.59E-03 | -0.173 | 6.90E-03 | -0.134 | 9.42E-05 | 0.467 | 0 |
| rs11644741 | 16 | 75466433 | C/A | *CFDP1* | -0.116 | 4.07E-03 | -0.182 | 4.07E-03 | -0.135 | 7.35E-05 | 0.376 | 0 |
| rs12917651 | 16 | 75466696 | G/T | *CFDP1* | -0.118 | 3.59E-03 | -0.182 | 4.07E-03 | -0.137 | 6.32E-05 | 0.394 | 0 |
| rs35787595 | 16 | 75466847 | G/C | *CFDP1* | -0.118 | 3.59E-03 | -0.183 | 4.02E-03 | -0.137 | 6.25E-05 | 0.392 | 0 |
| rs12929673 | 16 | 75470295 | C/T | *CFDP1* | -0.116 | 4.07E-03 | -0.178 | 4.97E-03 | -0.134 | 8.45E-05 | 0.408 | 0 |
| rs35209155 | 16 | 75470496 | G/A | *CFDP1* | -0.116 | 4.07E-03 | -0.178 | 4.97E-03 | -0.134 | 8.45E-05 | 0.408 | 0 |
| rs8055974 | 16 | 75470986 | C/G | *CFDP1* | -0.109 | 6.65E-03 | -0.178 | 4.97E-03 | -0.129 | 1.47E-04 | 0.358 | 0 |
| rs4887825 | 16 | 75472422 | C/A | *CFDP1* | -0.109 | 6.65E-03 | -0.178 | 5.10E-03 | -0.129 | 1.50E-04 | 0.361 | 0 |
| rs34021527 | 16 | 75474090 | A/G | *CFDP1* | -0.109 | 7.15E-03 | -0.177 | 5.19E-03 | -0.129 | 1.62E-04 | 0.364 | 0 |
| rs56004344 | 16 | 75474189 | T/C | *CFDP1* | -0.109 | 7.15E-03 | -0.177 | 5.19E-03 | -0.129 | 1.62E-04 | 0.364 | 0 |
| rs7204984 | 16 | 75475520 | G/C | *CFDP1* | -0.108 | 7.77E-03 | -0.171 | 7.70E-03 | -0.126 | 2.39E-04 | 0.406 | 0 |
| rs35214308 | 16 | 75476088 | C/T | *CFDP1* | -0.105 | 8.70E-03 | -0.171 | 7.70E-03 | -0.124 | 2.74E-04 | 0.389 | 0 |
| rs4888425 | 16 | 75476363 | T/C | *CFDP1* | -0.108 | 7.77E-03 | -0.171 | 7.70E-03 | -0.126 | 2.39E-04 | 0.406 | 0 |
| rs4888426 | 16 | 75476451 | G/A | *CFDP1* | -0.108 | 7.77E-03 | -0.171 | 7.70E-03 | -0.126 | 2.39E-04 | 0.406 | 0 |
| rs2161684 | 16 | 75479153 | T/C | *CFDP1* | -0.108 | 7.77E-03 | -0.157 | 1.50E-02 | -0.122 | 3.90E-04 | 0.515 | 0 |
| rs35683383 | 16 | 75479277 | G/T | *CFDP1* | -0.112 | 5.92E-03 | -0.150 | 1.96E-02 | -0.122 | 3.53E-04 | 0.612 | 0 |
| rs34996006 | 16 | 75479434 | A/G | *CFDP1* | -0.108 | 7.77E-03 | -0.157 | 1.50E-02 | -0.122 | 3.90E-04 | 0.515 | 0 |
| rs12927562 | 16 | 75482860 | G/A | *CFDP1* | -0.105 | 8.70E-03 | -0.157 | 1.50E-02 | -0.120 | 4.44E-04 | 0.496 | 0 |
| rs14050 | 17 | 37828072 | C/T | *PGAP3* | -0.332 | 3.97E-15 | -0.222 | 9.13E-04 | -0.301 | 4.02E-17 | 0.163 | 48 |
| rs732083 | 17 | 37834367 | G/A | *PGAP3* | -0.351 | 2.66E-18 | -0.198 | 2.58E-03 | -0.309 | 1.97E-19 | 0.047 | 75 |
| rs14050 | 17 | 37828072 | C/T | *GSDMB* | -0.099 | 2.31E-02 | -0.152 | 2.59E-03 | -0.121 | 2.27E-04 | 0.422 | 0 |

*β*_GTEx_ and *β*_TCGA_, eQTL effect size in GTEx pancreas and TCGA pancreatic tumor, respectively; *P*_GTEx_ and *P*_TCGA_, corresponding eQTL *P* value; *β*_meta_, effect size from the fixed-effects inverse-variance meta-analysis; *P*_meta_, corresponding meta-analysis *P* value; *I*^2^, percentage of total variation across studies due to heterogeneity; *P*_Het_, *P* value for heterogeneity (Cochran’s Q test).

**Table S5.** Regulatory annotation of candidate SNPs through eQTL-GWAS integration.

| SNP | RegulomeDB Score | CADD PHRED Score | Epigenetic Marks |
| --- | --- | --- | --- |
| rs11102484 | 2b | 9.64 | H3K4me3, DHS, H3K4me1, H3K27ac, H3K27me3, H3K9me3, H3K36me3 |
| rs2161648 | 2b | 6.70 | H3K4me1, H3K4me3, H3K27ac, H3K27me3, H3K9me3, H3K36me3 |
| rs11862684 | 2b | 0 | DHS, H3K4me1, H3K36me3 |
| rs4888372 | 3a | 8.07 | ATAC-seq, H3K4me1, H3K4me3, DHS, H3K27ac, H3K27me3, H3K9me3, H3K36me3 |
| rs3000075 | 3a | 4.63 | H3K4me3, DHS, H3K4me1, H3K9me3, H3K36me3 |
| rs2816608 | 3a | 4.61 | H3K4me3, DHS, H3K4me1, H3K27ac, H3K27me3, H3K9me3, H3K36me3 |
| rs35683383 | 3a | 3.48 | H3K4me1, H3K4me3, H3K27ac, H3K27me3, H3K9me3, H3K36me3 |
| rs6714988 | 3a | 3.10 | H3K4me3, H3K4me1, H3K27ac, H3K27me3, H3K9me3, H3K36me3 |
| rs12930452 | 3a | 2.84 | DHS, H3K4me1, H3K27ac, H3K36me3 |
| rs612045 | 3a | 2.43 | DHS, H3K27me3 |
| rs6739471 | 3b | 2.30 | H3K4me1, H3K9me3 |
| rs6624 | 4 | 9.89 | H3K4me3, H3K4me1, H3K27ac, H3K27me3, H3K9me3, H3K36me3 |
| rs14050 | 4 | 8.91 | H3K4me3, DHS, H3K4me1, H3K27ac, H3K27me3, H3K9me3, H3K36me3 |
| rs3828074 | 4 | 8.29 | H3K4me3, H3K4me1, DHS, H3K27ac, H3K27me3, H3K9me3, H3K36me3 |
| rs3790605 | 4 | 7.58 | H3K4me3, DHS, H3K4me1, H3K27ac, H3K9me3, H3K36me3 |
| rs12136566 | 4 | 6.63 | H3K4me3, H3K27ac, H3K4me1, DHS, H3K9me3, H3K36me3 |
| rs2735817 | 4 | 5.80 | H3K4me3, H3K4me1, H3K27ac, H3K9me3, H3K36me3 |
| rs35787595 | 4 | 4.84 | H3K4me3, H3K27ac, DHS, H3K4me1, H3K27me3, H3K9me3, H3K36me3 |
| rs594422 | 4 | 4.63 | H3K4me3, DHS, H3K4me1, H3K27ac, H3K36me3 |
| rs12929908 | 4 | 3.60 | H3K4me1, H3K27ac, H3K36me3 |
| rs732083 | 4 | 2.95 | H3K4me1, H3K27ac, H3K4me3, H3K27me3, H3K9me3, H3K36me3 |
| rs11149833 | 4 | 2.30 | H3K4me1, H3K27ac, H3K27me3, H3K9me3, H3K36me3 |
| rs12928898 | 4 | 2.23 | DHS, H3K4me1, H3K27ac, H3K36me3 |
| rs595971 | 4 | 2.07 | H3K4me3, DHS, H3K4me1, H3K27ac, H3K27me3, H3K9me3, H3K36me3 |
| rs12917651 | 4 | 1.58 | H3K4me3, H3K27ac, DHS, H3K4me1, H3K27me3, H3K9me3, H3K36me3 |
| rs11644741 | 4 | 1.57 | H3K4me3, H3K4me1, DHS, H3K27ac, H3K27me3, H3K9me3, H3K36me3 |
| rs35209155 | 4 | 1.30 | H3K4me3, H3K4me1, H3K27ac, H3K27me3 |
| rs7146643 | 4 | 0.87 | H3K4me3, DHS, H3K4me1, H3K27ac, H3K9me3, H3K36me3 |
| rs12747682 | 4 | 0.75 | H3K4me1, H3K4me3, DHS, H3K27ac, H3K9me3, H3K36me3 |
| rs596375 | 4 | 0.44 | H3K4me3, H3K4me1, H3K27ac, H3K9me3, H3K36me3 |
| rs596474 | 4 | 0.23 | H3K4me3, H3K4me1, H3K27ac, H3K9me3, H3K36me3 |
| rs12445726 | 4 | 0.19 | H3K4me1, H3K4me3, DHS, H3K27ac, H3K9me3, H3K36me3 |
| rs34996006 | 4 | 0.17 | H3K4me1, H3K4me3, DHS, H3K27ac, H3K27me3, H3K9me3, H3K36me3 |
| rs2816632 | 4 | 0.17 | H3K4me3, DHS, H3K4me1, H3K27ac, H3K27me3, H3K9me3, H3K36me3 |
| rs4888418 | 4 | 0.07 | DHS, H3K4me1, H3K27ac, H3K9me3, H3K36me3 |
| rs12929673 | 4 | 0.06 | H3K4me3, DHS, H3K4me1, H3K27me3, H3K9me3, H3K36me3 |
| rs4888426 | 5 | 11.87 | H3K4me3, DHS, H3K4me1, H3K27ac, H3K27me3, H3K36me3 |
| rs1011121 | 5 | 10.83 | H3K4me3, DHS, H3K4me1, H3K27ac, H3K27me3, H3K9me3, H3K36me3 |
| rs1483351 | 5 | 10.58 | H3K4me3, H3K4me1, H3K27ac, H3K27me3, H3K36me3 |
| rs11861810 | 5 | 10.29 | H3K4me3, DHS, H3K4me1, H3K27ac, H3K27me3, H3K9me3, H3K36me3 |
| rs11149832 | 5 | 10.11 | H3K4me1, H3K27me3, H3K9me3, H3K36me3 |
| rs7204984 | 5 | 6.49 | H3K4me3, H3K4me1, H3K27ac, H3K27me3, H3K9me3, H3K36me3 |
| rs1733483 | 5 | 5.33 | H3K4me3, DHS, H3K4me1, H3K27ac, H3K27me3, H3K9me3, H3K36me3 |
| rs4887825 | 5 | 4.27 | H3K4me1 |
| rs11890682 | 5 | 4.18 | H3K4me3, DHS, H3K4me1, H3K27me3, H3K9me3, H3K36me3 |
| rs203424 | 5 | 4.13 | H3K4me3, H3K4me1, H3K27ac, H3K27me3, H3K9me3, H3K36me3 |
| rs619396 | 5 | 3.62 | H3K4me3, DHS, H3K4me1, H3K27ac, H3K27me3, H3K9me3, H3K36me3 |
| rs4888425 | 5 | 3.42 | H3K4me3, H3K4me1, H3K27ac, H3K27me3, H3K9me3, H3K36me3 |
| rs12930768 | 5 | 3.11 | H3K4me1, H3K4me3, DHS, H3K27ac, H3K27me3, H3K9me3, H3K36me3 |
| rs23506 | 5 | 2.77 | DHS |
| rs9934007 | 5 | 2.09 | DHS, H3K4me1, H3K27ac, H3K27me3, H3K36me3 |
| rs6546300 | 5 | 2.02 | H3K4me3, DHS, H3K4me1, H3K27ac, H3K27me3, H3K9me3, H3K36me3 |
| rs12448947 | 5 | 1.99 | DHS, H3K4me1, H3K27ac, H3K27me3, H3K9me3, H3K36me3 |
| rs2161684 | 5 | 1.90 | DHS, H3K4me1, H3K27ac, H3K27me3, H3K36me3 |
| rs11646044 | 5 | 1.21 | DHS, H3K4me1, H3K27ac, H3K27me3, H3K9me3, H3K36me3 |
| rs11641587 | 5 | 1.10 | H3K4me1, DHS, H3K27ac, H3K9me3, H3K36me3 |
| rs6546298 | 5 | 0.96 | H3K4me3, H3K4me1, H3K27ac, H3K9me3, H3K36me3 |
| rs12449170 | 5 | 0.91 | H3K4me1, H3K27ac, H3K27me3, H3K36me3 |
| rs4888420 | 5 | 0.86 | DHS, H3K4me1, H3K27ac, H3K27me3, H3K36me3 |
| rs12927562 | 5 | 0.40 | H3K4me3, DHS, H3K4me1, H3K27ac, H3K27me3, H3K9me3, H3K36me3 |
| rs13246232 | 5 | 0.37 | H3K4me3, DHS, H3K4me1, H3K27me3, H3K9me3, H3K36me3 |
| rs34021527 | 5 | 0.28 | DHS, H3K4me1, H3K27ac, H3K27me3, H3K9me3, H3K36me3 |
| rs35214308 | 5 | 0.19 | H3K4me3, DHS, H3K4me1, H3K27ac, H3K27me3, H3K9me3, H3K36me3 |
| rs4594277 | 5 | 0.10 | H3K4me1, H3K27ac, H3K9me3, H3K36me3 |
| rs8055974 | 5 | 0.06 | H3K4me1, H3K27ac, H3K27me3, H3K9me3, H3K36me3 |
| rs6732641 | 5 | 0.05 | H3K4me3, DHS, H3K4me1, H3K27ac, H3K9me3, H3K36me3 |
| rs622444 | 5 | 0.03 | H3K4me3, H3K4me1, H3K27ac, H3K9me3, H3K36me3 |
| rs606589 | 6 | 6.39 | H3K4me1 |
| rs7194129 | 6 | 3.29 | H3K4me3, H3K4me1, H3K27ac, H3K27me3, H3K9me3, H3K36me3 |
| rs9554197 | 6 | 2.40 | DHS, H3K4me1, H3K36me3 |
| rs7199680 | 6 | 2.39 | H3K4me3, H3K4me1, H3K27ac, H3K27me3, H3K9me3, H3K36me3 |
| rs7564335 | 6 | 2.09 | H3K4me1, H3K27ac, H3K27me3, H3K9me3, H3K36me3 |
| rs11618581 | 6 | 1.54 | DHS, H3K4me1, H3K27ac, H3K36me3 |
| rs56004344 | 6 | 0.26 | H3K4me1, H3K27ac, H3K27me3, H3K9me3, H3K36me3 |
| rs3770657 | 7 | 9.36 | DHS, H3K27ac, H3K36me3 |
| rs1032158 | 7 | 2.48 | H3K4me3, DHS, H3K4me1, H3K27ac, H3K9me3, H3K36me3 |
| rs1027000 | 7 | 1.75 | H3K4me3, H3K4me1, H3K27me3, H3K9me3, H3K36me3 |
| rs1601530 | 7 | 1.75 | H3K27me3, H3K36me3 |
| rs4887813 | 7 | 0.81 | DHS, H3K4me1, H3K27ac, H3K27me3, H3K9me3, H3K36me3 |
| rs670502 | 7 | 0.70 | H3K4me3, DHS, H3K4me1, H3K27ac, H3K27me3, H3K9me3, H3K36me3 |
| rs6564249 | 7 | 0.58 | H3K4me3, H3K4me1, H3K27ac, H3K36me3 |
| rs598637 | 7 | 0.33 | DHS |

**Table S6.** Demographic characteristics of cases and controls in the replication cohort.

| Variable | Cases | Controls | *P* |
| --- | --- | --- | --- |
| Age, mean ± SD, years | 58.70 ± 12.96 | 60.20 ± 10.18 | 0.010 |
| Gender, n (%) |  |  | 0.260 |
| Male | 304 (53.43%) | 1,510 (56.11%) |  |
| Female | 265 (46.57%) | 1,181 (43.89%) |  |
| Total | 569 | 2,691 |  |

SD, standard deviation.

Differences in age and gender distributions between cases and controls are assessed using Student’s *t*-test and the chi-square test, respectively.

**Table S7.** Oligonucleotides used in this study.

| siRNA |  |  |
| --- | --- | --- |
| *ST7L* (the target sequence) | siRNA#1 | GAACCAGGTTCTCCTTCGA |
|  | siRNA#2 | GCAGCACTGTTGAAGACAA |
| *ZNF263* | siRNA#1 | GGUGUGGACUGCACUGAAA |
|  | siRNA#2 | CGAGAGUCACCGAGCUUCA |
|  | siRNA#3 | CGAAAGAACUCAUGAGAGA |
| RT-qPCR (5’-3’) |  |  |
| *ST7L* | Forward | TCCATACCCGTTAGAGAAAGG |
|  | Reverse | GGCTATCATTGCTGTAGAAGAG |
| *GAPDH* | Forward | GGAGTCCACTGGCGTCTTCA |
|  | Reverse | GTCATGAGTCCTTCCACGATACC |
| Electrophoretic mobility shift assay (5’-3’) | |  |
| rs11102484-Ref | Forward | CTCGGGGATCCTCCTCCCGGGCTCT |
|  | Reverse | AGAGCCCGGGAGGAGGATCCCCGAG |
| rs11102484-Mut | Forward | CTCGGGGATCCTGCTCCCGGGCTCT |
|  | Reverse | AGAGCCCGGGAGCAGGATCCCCGAG |
| Chromatin immunoprecipitation qPCR assay (5’-3’) | | |
| The rs11102484-containing fragment | Forward | GGGGACAGGAGGGAAGTGCCCGCAT |
|  | Reverse | GGAGCAGAAGGCAGGCAAGACC |
| *ST7L* promoter | Forward | TATATTTTCCCAAGTTTAATTAAAG |
|  | Reverse | CCATCCGTCTTCGCCACTTA |
| CRISPR/Cas9 sgRNA | | |
| sgRNAs targeting the silencer | sgRNA-1 | GTGTGGCTCTCAGGCTGGGCGGG |
|  | sgRNA-2 | AGGCTGTTGCTTCGACGGGTTGG |

**
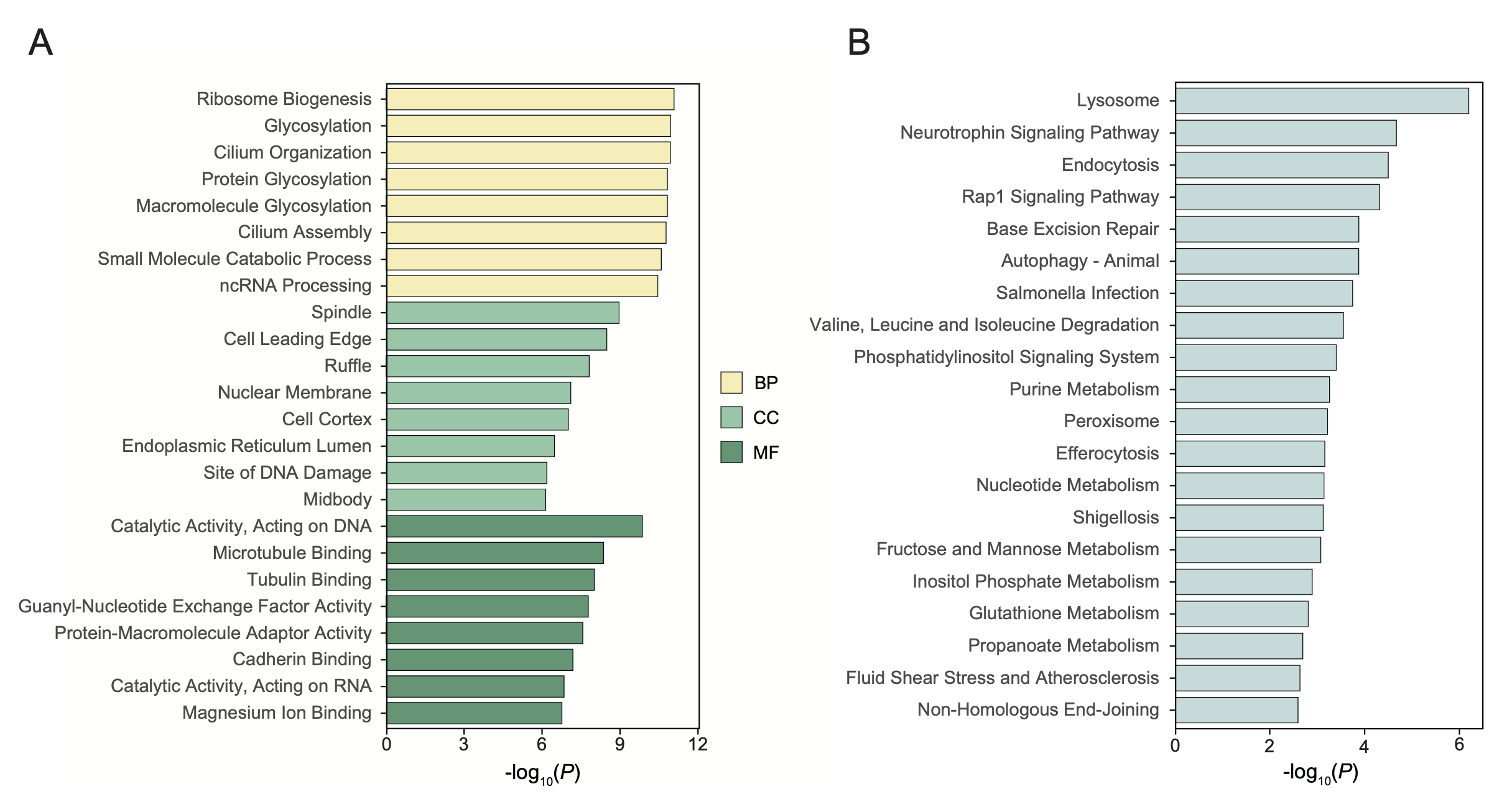
Supplementary figures**

**Figure S1.** GO and KEGG enrichment analysis of eGenes. A) GO enrichment of eGenes across three categories: biological process (BP), cellular component (CC), and molecular function (MF). The top eight significantly enriched terms in each category are shown, ranked by -log_10_(*P*). B) KEGG pathway enrichment of eGenes. The top 20 significantly enriched pathways are displayed, ranked by -log_10_(*P*). Enrichment is assessed using a hypergeometric test, and *P* values are adjusted using the BH method.

**
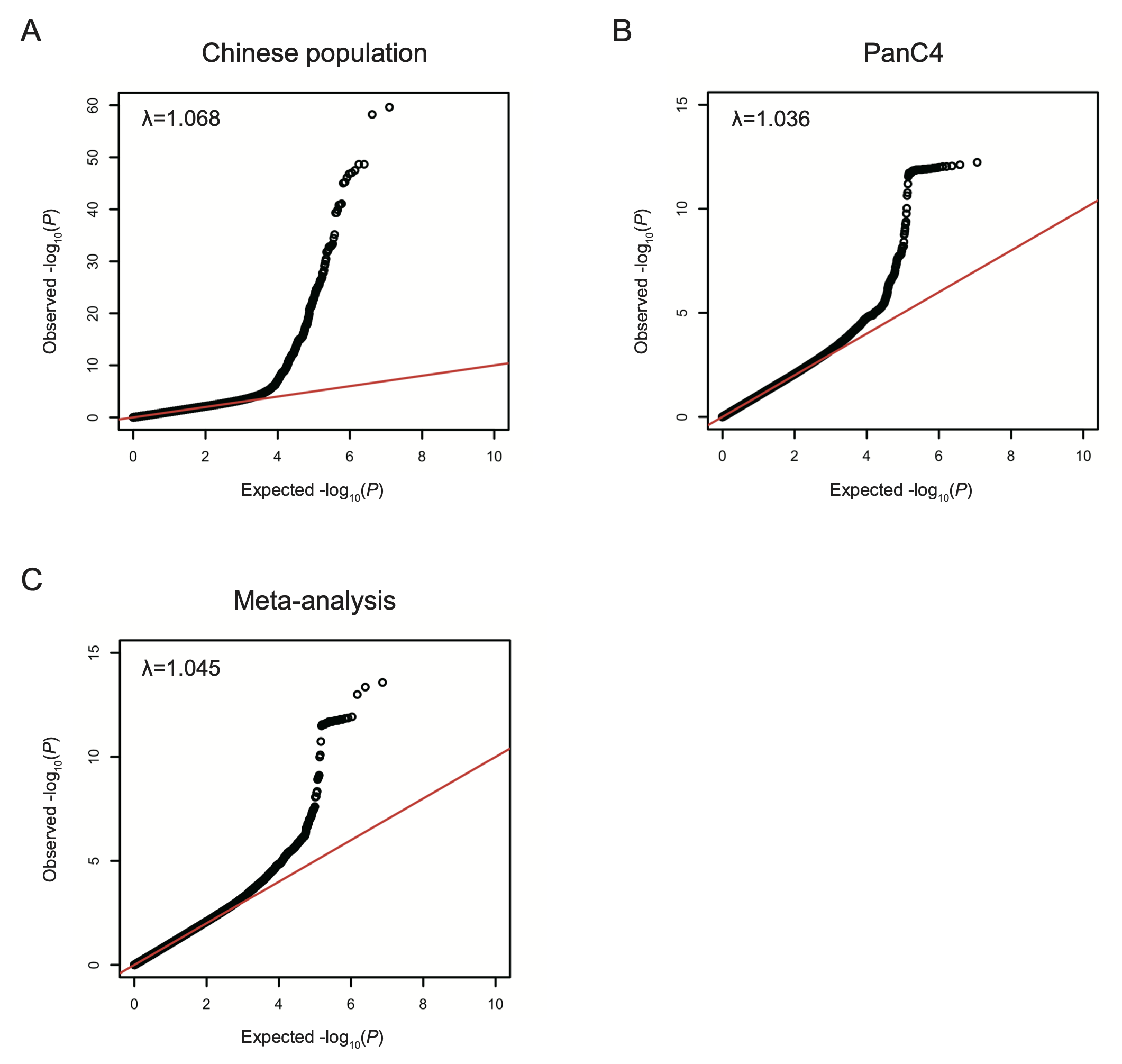
****Figure S2.** Quantile-quantile (Q-Q) plots of association *P* values. A-C) Q-Q plots of observed versus expected *P* values from association analyses after genotype imputation in the Chinese GWAS cohort (A), the PanC4 cohort (B), and the combined meta-analysis (C).

**
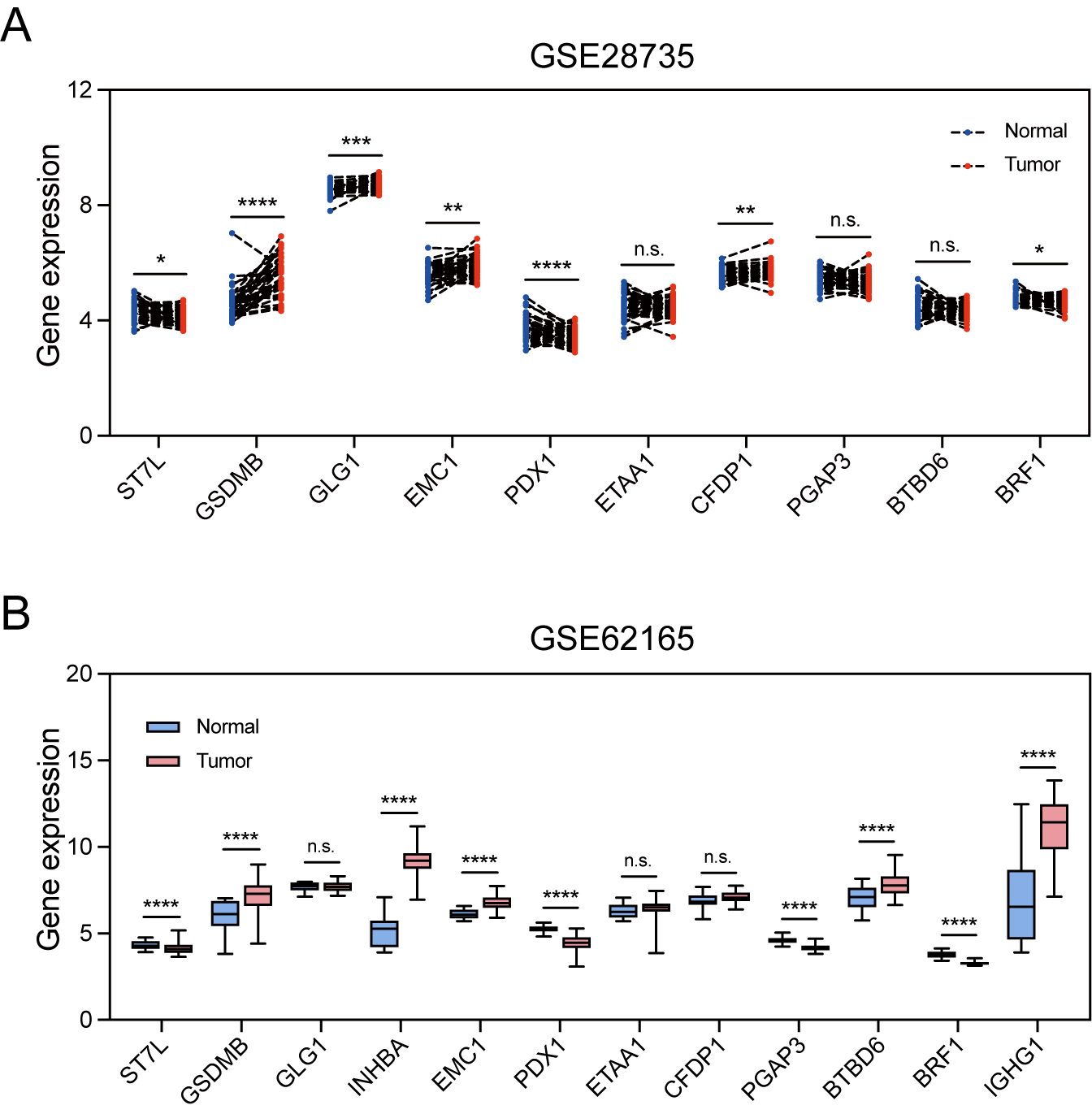
**

**Figure S3.** Differential expression of prioritized candidate genes in pancreatic cancer datasets. A,B) Differential expression analysis between pancreatic tumor and control tissues in GSE28735 (*n* = 45 pairs) (A) and GSE62165 (tumor tissues *n* = 118; normal tissues *n* = 13) (B). Data are analyzed using limma with empirical Bayes moderation (a paired model for GSE28735; a two-group model for GSE62165). *P* values are adjusted using the BH method (FDR). n.s., not significant; *FDR < 0.05; **FDR < 0.01; ***FDR < 0.001; ****FDR < 0.0001.

**
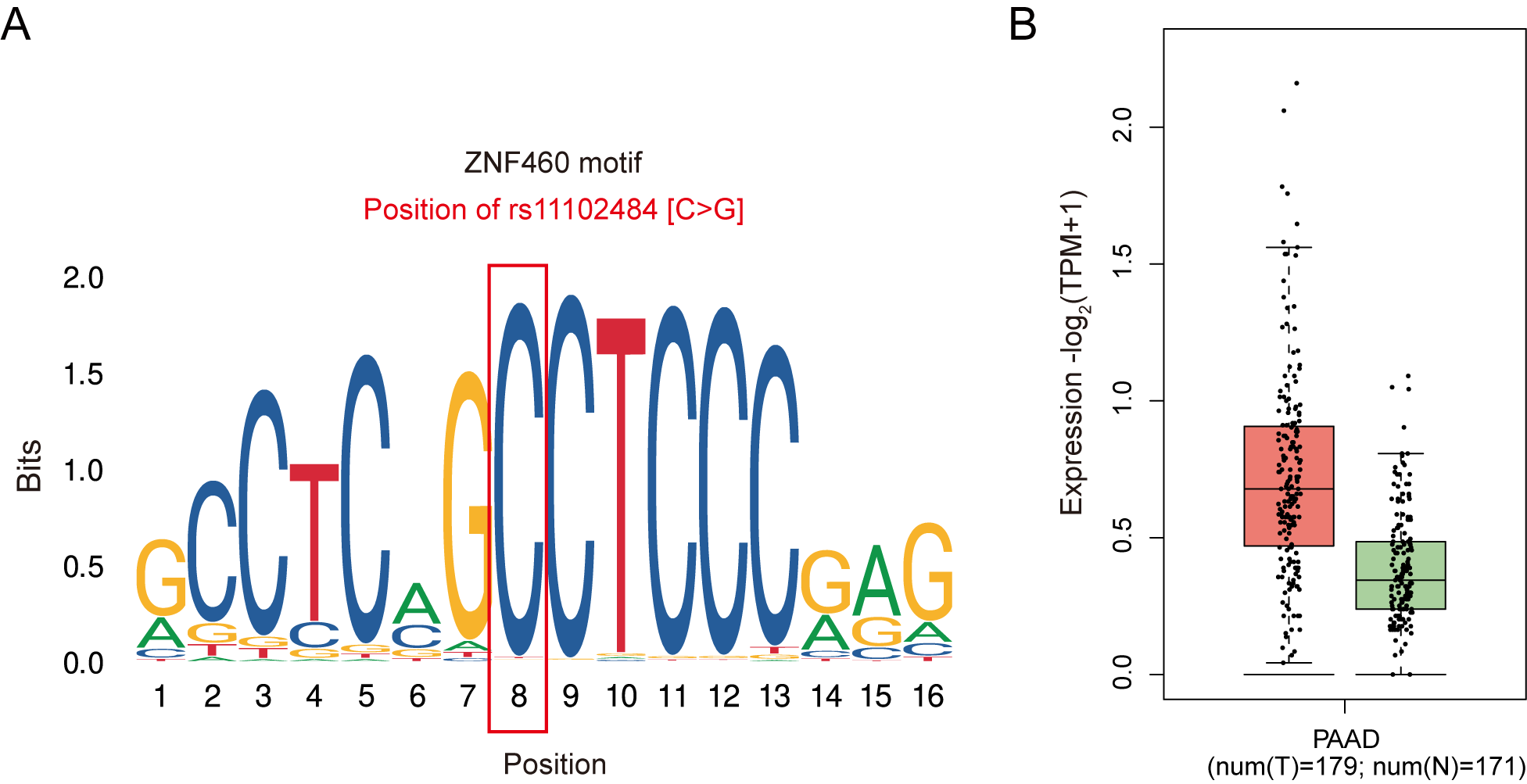
**

**Figure S4.** Characterization of the ZNF460 motif and its expression pattern. A) Predicted ZNF460 binding motif from JASPAR. The position corresponding to rs11102484 is highlighted by a red box. B) *ZNF460* shows no significant differential expression between pancreatic cancer (*n* = 179) and normal (*n* = 171) tissues based on GEPIA. Red, tumor (T); green, normal (N).

**
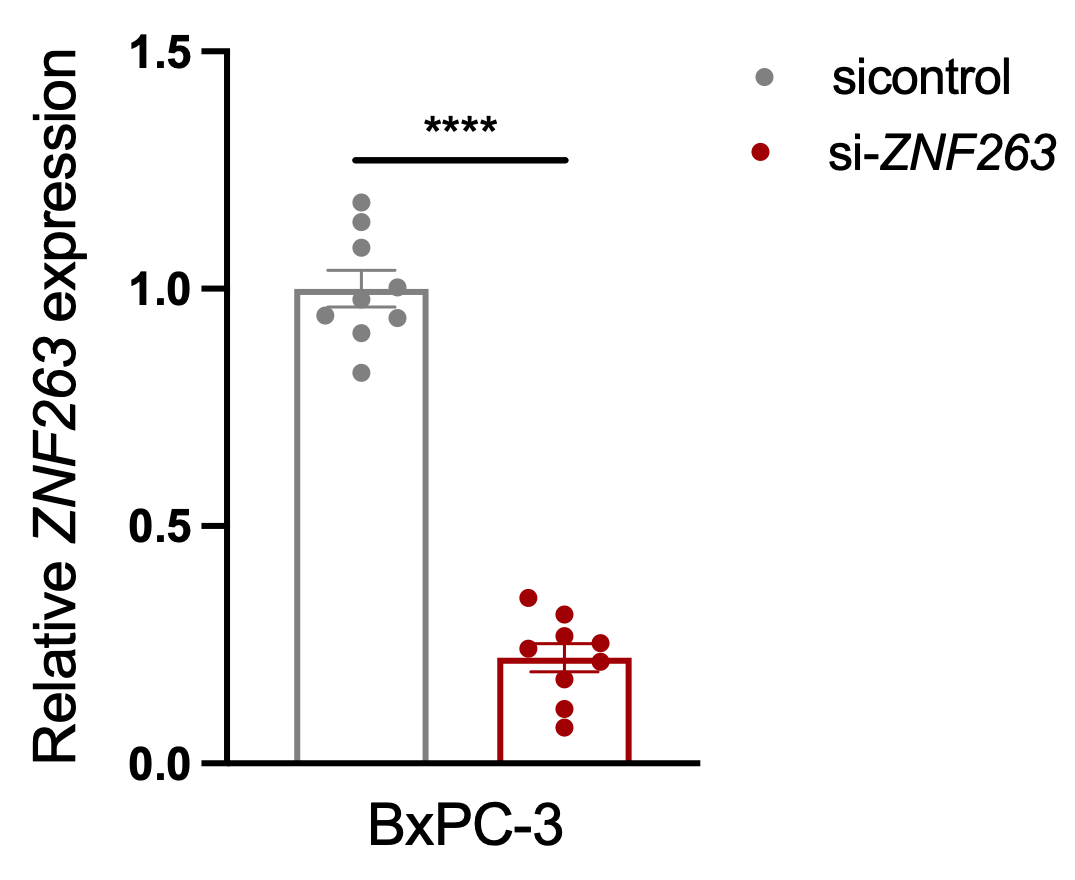
**

**Figure S5.** *ZNF263* expression after knockdown in BxPC-3 cells, as measured by RT-qPCR. Error bars represent mean ± s.e.m. from *n* = 3 independent experiments, each performed with three technical replicates. *****P* < 0.0001 (two-tailed unpaired Student’s *t*-test).

**
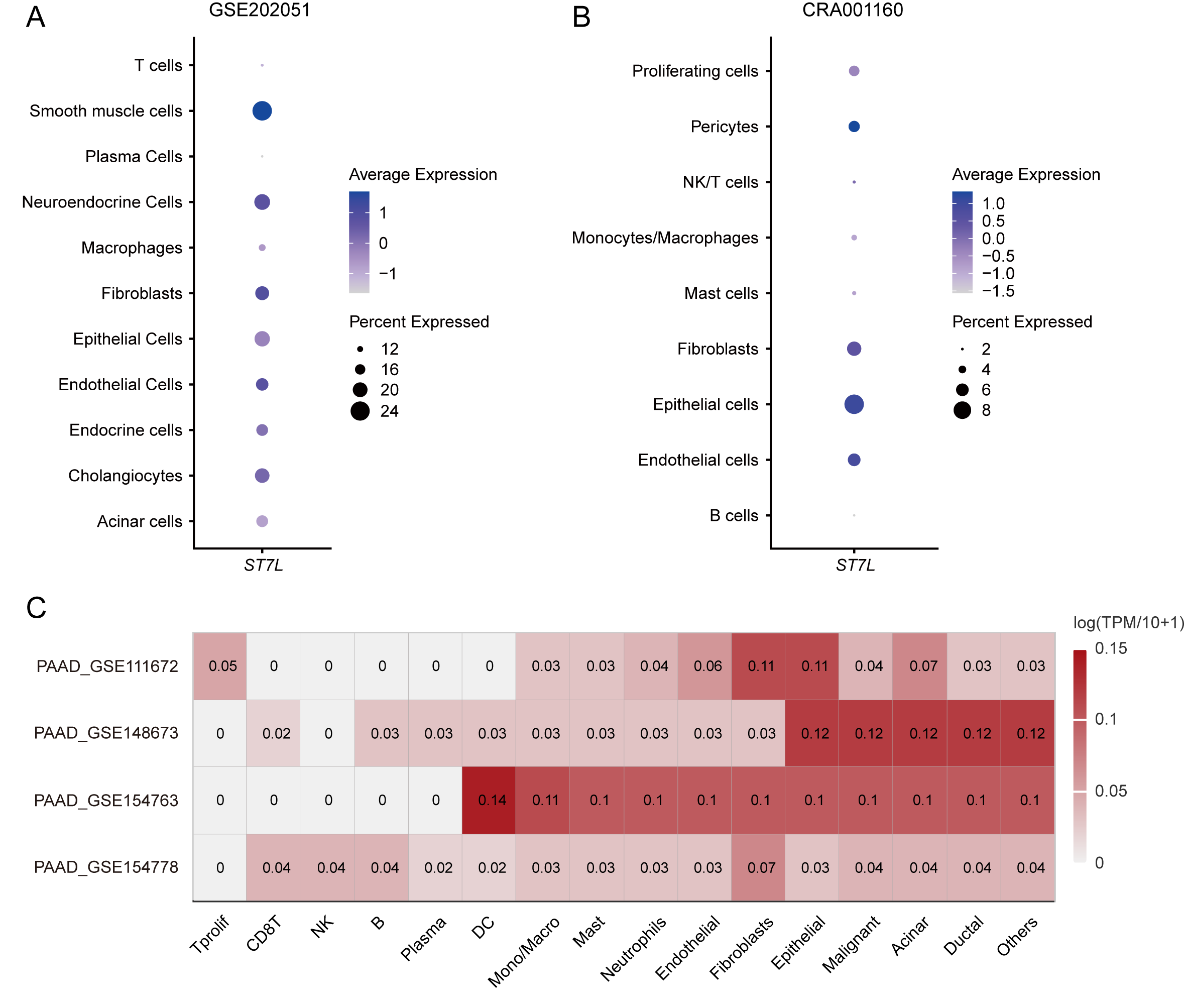
****Figure S6.** Expression pattern of *ST7L* across different cell types in pancreatic cancer. A,B) Bubble plots showing *ST7L* expression across major cell types, based on the snRNA-seq dataset GSE202051 from GEO (A) and scRNA-seq dataset CRA001160 from GSA (B). Bubble size indicates the percentage of *ST7L*-positive cells within each cell type, and color denotes scaled average expression (z-score). C) Heatmap summarizing *ST7L* expression across annotated cell types in curated pancreatic cancer single-cell datasets (GSE111672, GSE148673, GSE154763, and GSE154778) from the TISCH2 database.

**
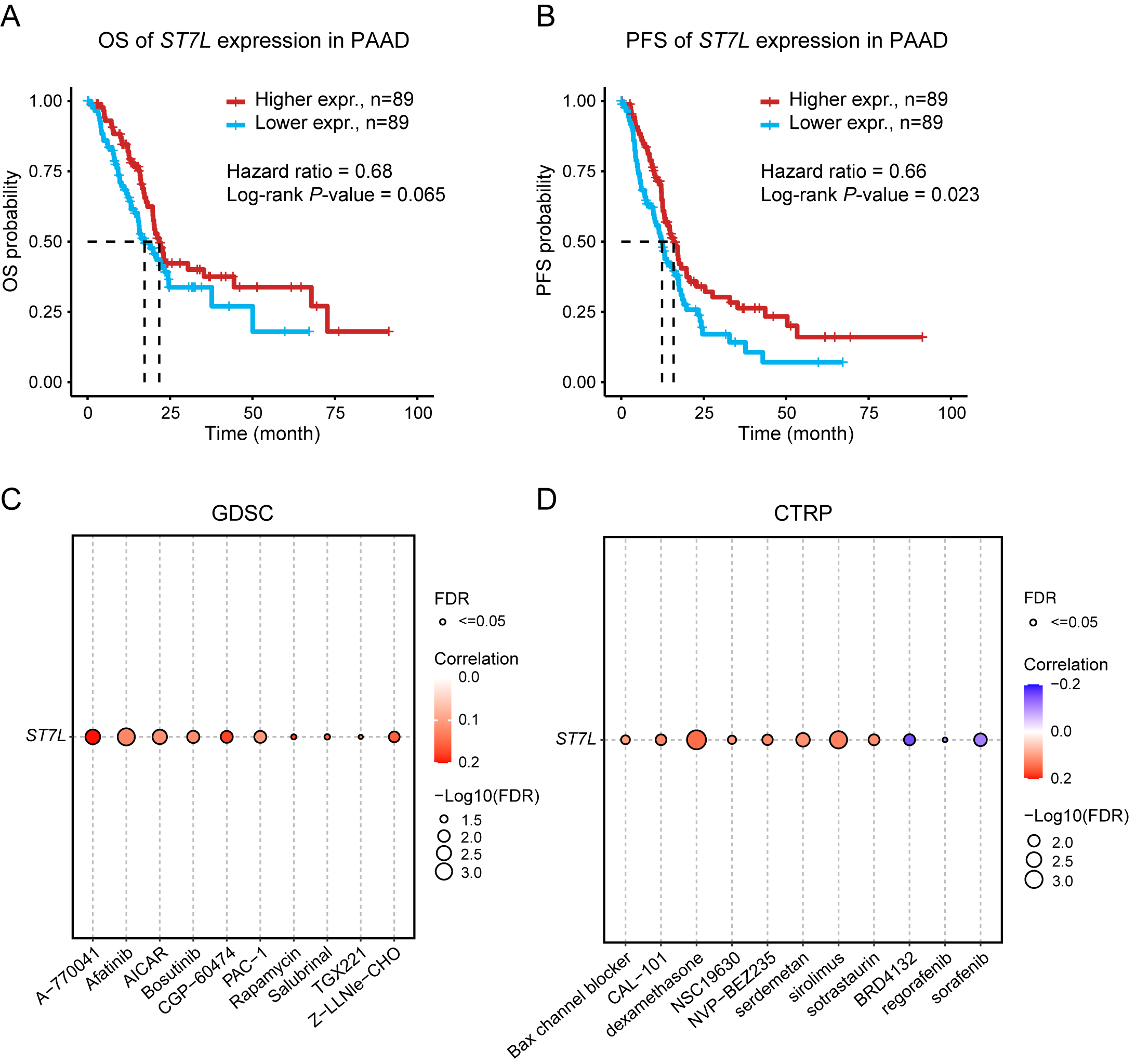
Figure S7.** Clinical relevance and drug sensitivity associations of *ST7L*. A,B) Kaplan-Meier curves of overall survival (A) and progression-free survival (B) for patients in the TCGA pancreatic cancer cohort, generated using the GSCA platform. Patients are stratified into high- and low-*ST7L* expression groups according to the median expression level. *P* values are calculated using the log-rank test. OS, overall survival; PFS, progression-free survival; expr., expression. C, D) Correlations between *ST7L* mRNA expression and drug sensitivity in cancer cell lines based on GDSC (C) and CTRP (D) datasets, as analyzed using the GSCA platform. Each bubble represents the correlation of *ST7L* expression with the IC50 of an individual drug. Bubble size reflects -log_10_(FDR), and color indicates the Pearson correlation coefficient.


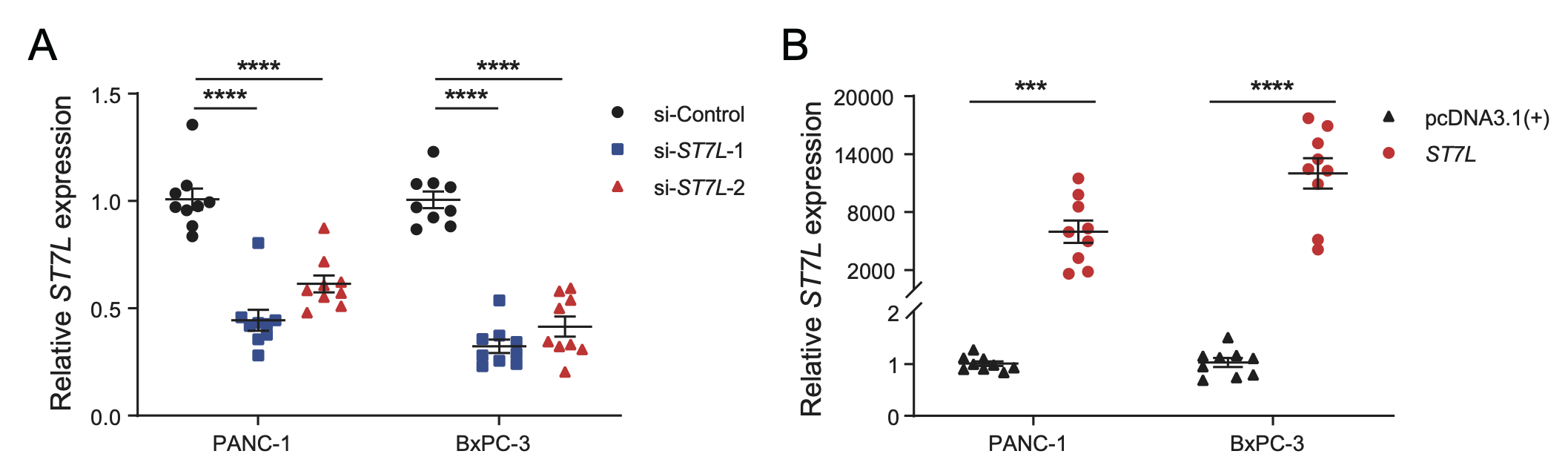
**Figure S8.** *ST7L* expression after knockdown or overexpression. A,B) Relative expression levels of *ST7L* in PANC-1 and BxPC-3 cells measured by RT-qPCR following knockdown (A) or overexpression (B). Error bars represent mean ± s.e.m. from *n* = 3 independent experiments, each performed with three technical replicates. ****P* < 0.001; *****P* < 0.0001 (two-tailed unpaired Student’s *t*-test).
